# Risk of hematologic malignancies after breast ductal carcinoma in situ treatment with ionizing radiation

**DOI:** 10.1038/s41523-021-00228-6

**Published:** 2021-03-02

**Authors:** Kang Wang, Zhuyue Li, Xingxing Chen, Jianjun Zhang, Yongfu Xiong, Guochao Zhong, Yang Shi, Qing Li, Xiang Zhang, Hongyuan Li, Tingxiu Xiang, Theodoros Foukakis, Tomas Radivoyevitch, Guosheng Ren

**Affiliations:** 1 Department of Endocrine and Breast Surgery, The First Affiliated hospital of Chongqing Medical University, Chongqing Medical University, Chongqing, China; 2 Key Laboratory of Molecular Oncology and Epigenetics, The First Affiliated Hospital of Chongqing Medical University, Chongqing, China; 3 Department of Oncology-Pathology, Karolinska Institutet, Stockholm, Sweden; 4 West China Hospital/West China School of Nursing, Sichuan University, Chengdu, China; 5 Department of Oncology, Shanghai Medical College, Fudan University, Shanghai, China; 6 Department of Radiation Oncology, Fudan University Shanghai Cancer Center, Shanghai, China; 7 Department of Epidemiology, Fairbanks School of Public Health and Melvin and Bren Simon Comprehensive Cancer Center, Indiana University, 1050 Wishard Boulevard RG5118, Indianapolis, IN USA; 8 The First Department of Hepatobiliary Surgery, Affiliated Hospital of North Sichuan Medical College, Nanchong, China; 9 Department of Hepatobiliary Surgery, the Second Affiliated Hospital of Chongqing Medical University, Chongqing, China; 10 Division of Biostatistics and Data Science, Department of Population Health Sciences, Medical College of Georgia, Augusta University, Augusta, GA USA; 11 Breast Center, Theme Cancer, Karolinska University Hospital, Stockholm, Sweden; 12 Quantitative Health Sciences, Lerner Research Institute, Cleveland Clinic, Cleveland, OH USA

**Keywords:** Cancer epidemiology, Breast cancer, Risk factors

## Abstract

The increased incidence of secondary hematologic malignancies (SHM) is a well-known, potentially fatal, complication after cancer treatment. It is unknown if patients with ductal carcinoma in situ (DCIS) of the breast treated with external beam radiotherapy (RT) and who survive long-term have increased risks of secondary hematologic malignancies (SHM), especially for low/intermediate-risk subsets with limited benefits from RT. DCIS patients in Surveillance, Epidemiology, and End Results (SEER) registries (1975–2016) were identified. Relative risks (RR), hazard ratio (HR), and standardized incidence ratios (SIR) were calculated to assess the SHM risk and subsequent survival times. SHM development, defined as a nonsynchronous SHM occurring ≥1 year after DCIS diagnosis, was our primary endpoint. Of 184,363 eligible patients with DCIS, 77,927 (42.3%) in the RT group, and 106,436 (57.7%) in the non-RT group, 1289 developed SHMs a median of 6.4 years (interquartile range, 3.5 to 10.3 years) after their DCIS diagnosis. Compared with DCIS patients in the non-RT group, RT was associated with increased early risk of developing acute lymphoblastic leukemia (ALL; hazard ratio, 3.15; 95% CI, 1.21 to 8.17; *P* = 0.02), and a delayed risk of non-Hodgkin lymphoma (NHL; hazard ratio, 1.33; 95% CI, 1.09 to 1.62; *P* < 0.001). This increased risk of ALL and NHL after RT was also observed in subgroup analyses restricted to low/intermediate-risk DCIS. In summary, our data suggest that RT after breast conserving surgery for DCIS patients should be cautiously tailored, especially for low and intermediate-risk patients. Long-term SHM surveillance after DCIS diagnosis is warranted.

## Introduction

It has been estimated that over 48,000 new cases of ductal carcinoma in situ (DCIS) of the breast will be diagnosed in the United States in 2019^1,2^, largely due to early detection by mammography^3^. DCIS, regarded as a true (nonobligatory) precursor lesion for invasive cancer, has an excellent prognosis—breast cancer-specific survival exceeds 95% after 15 year-follow-up^4,5^, resulting in a large number of DCIS survivors^3^. Treatment for DCIS usually involves either breast conserving surgery (BCS) with radiotherapy (RT) or mastectomy, where RT after BCS reduces the risk of ipsilateral local recurrence^6,7^. The potential improvement in survival offered by RT differs on the basis of patient factors, tumor biology, and the prognostic score^8^. Several prognostic score systems^9–11^ have been developed to assess risk of recurrence for DCIS using age at diagnosis, tumor size, nuclear grade, and surgical margin status, which have also been used to individualize RT administration^8,12–14^. RT associates with increases in the incidence of second primary malignancies in DCIS patients^15–18^.

Exposure to external beam RT for the first primary cancer is a well-established risk factor for secondary hematologic malignancies (SHMs)^19–22^, but previous studies often group all types of SHMs under broad leukemia and lymphoma categories when studying DCIS^16–18,23^, ignoring the biologic heterogeneity and disparate natural history of SHM subtypes. Striking differences have been documented for the incidence, latency period, treatments and outcomes of distinct SHMs^20,24–29^. SHMs can go undetected if there are few patients and short life expectancies^24,30^. In contrast, DCIS is a common disease and patients have almost normal life expectancies after adequate treatment, thus the risk of developing SHMs after bone marrow exposure to radiation should be considered. These exposures are especially relevant for low-/intermediate-risk patients for whom the benefit from RT is limited. Therefore, we sought to investigate the risk of developing SHMs including acute and chronic leukemias, lymphomas, and multiple myeloma in patients with DCIS treated with RT, and subsequent survival times.

## Results

### Patient characteristics

**Fig. 1:**
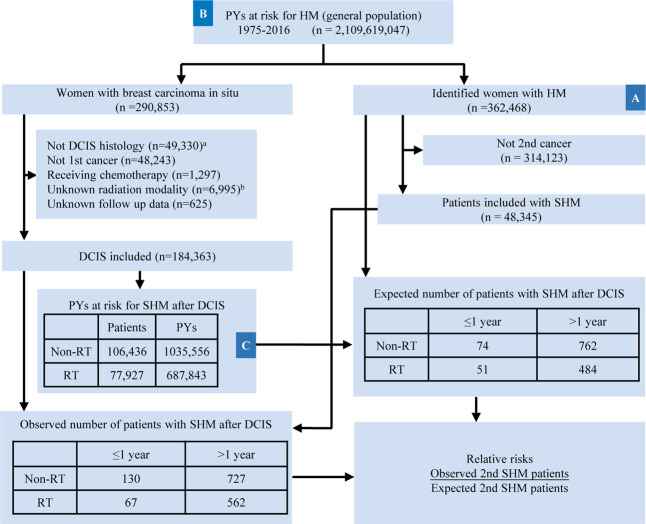
Population-based assessments of second hematologic malignancy (SHM) risks after ductal carcinoma in situ (DCIS) of the breast. SEER covers an increasing proportion of the US population, 2.11 billion person-years (PYs) since 1975. Shown is a flowchart of the inclusion of patients with DCIS and SHM and their use in calculations of relative risks (RRs) of SHM occurrence after DCIS. RRs are the number of observed patients with SHM after DCIS divided by the number of expected patients with SHM after DCIS. The latter is calculated using background incidence rate of SHM per PY, which is formed by dividing the number of hematologic malignancy (HM) patients by (**a**) the number of PYs at risk in the general population (**b**). Calculations account for age, sex, and year of diagnosis. Background incidence were multiplied by **c**, the PYs at risk among DCIS survivors in these demographic cohorts, to obtain the expected number of patients with SHMs after DCIS. In boxes titled “Expected patients with SHM after DCIS” numbers shown are expected numbers of patients with SHM diagnosed, ≤1 year or, >1 year after DCIS diagnosis, by RT. Here RT = radiotherapy and y = year. ^a^Exclusion of patients with other histological tumor, Paget disease or DCIS with micro-invasion. ^b^Exclusion of patients with unknown radiation status, method or source of radiation unspecified, or patients who received radioisotopes or radioactive implant.

After identifying patients with DCIS, we also excluded patients whose radiation treatment method, or source, was unknown, and those subjects who received radioisotopes or radioactive implant (Fig. 1, Supplementary Tables 1 and 2). Of 184,363 eligible DCIS patients identified from 290,853 breast carcinoma in situ cases in the SEER database, 77,927 (42.3%) were in the RT group and 106,436 (57.7%) were in the non-RT group. Clinicopathologic characteristics of patients with DCIS according to delivery of RT are listed in Table 1 and Supplementary Tables 2–5. During a median follow-up of 13.6 years for DCIS survivors, we identified 1,289 nonsynchronous SHMs, with 562 (43.6%) in the RT group and 727 (56.4%) in the Non-RT group.

**Table 1:** DICS patient characteristics by receipt of RT.

Characteristic	Non-RT (*n* = 106,436)	RT (*n* = 77,927)	*P*
Median age at DCIS diagnosis, (IQR), years	58 (49, 69)	58 (50, 66)	<0.001^M^
Median year of DCIS diagnosis (IQR)	2006 (2000, 2011)	2008 (2002, 2012)	<0.001^M^
Race			
White	82,954 (77.9)	60,719 (77.9)	<0.001^χ^
Black	11,021 (10.4)	8414 (10.8)	
Others	12,461 (11.7)	8794 (11.3)	
Tumor size, mm			
1–9	32,331 (30.4)	27,625 (35.4)	<0.001^χ^
10–19	16,426 (15.4)	16,830 (21.6)	
20–49	15,018 (14.1)	10,283 (13.2)	
50+	5936 (5.6)	1748 (2.2)	
Unknown	36,725 (34.5)	21,441 (27.5)	
Grade			
I	12,238 (11.5)	8094 (10.4)	<0.001^χ^
II	32,123 (30.2)	26,038 (33.4)	
III	32,690 (30.7)	29,910 (38.4)	
Unknown	29,385 (27.6)	13,885 (17.8)	
ER			
Negative	8993 (8.4)	7676 (9.9)	<0.001^χ^
Positive	48,665 (45.7)	45,243 (58.1)	
Unknown	48,778 (45.8)	25,008 (32.1)	
PR			
Negative	13,530 (12.7)	11,921 (15.3)	<0.001^χ^
Positive	40,054 (37.6)	37,474 (48.1)	
Unknown	52,852 (49.7)	28,532 (36.6)	
Surgery			
No	4355 (4.1)	389 (0.5)	<0.001^χ^
BCS	46,670 (43.8)	76,054 (97.6)	
Mastectomy	43,529 (40.9)	1199 (1.5)	
Unknown	11,882 (11.2)	285 (0.4)	
Median follow-up time of DCIS (IQR), years	8.4 (3.9, 14.1)	8 (3.8, 12.8#)	<0.001^M^
Total person-years at risk	1035,556	687,843	

Data presented as No. (%) unless otherwise stated where percentages were calculated within rows. *P*-values were calculated using the Pearson Chi-Square test (*χ*) and Mann–Whitney U tests (M).

*IQR* interquartile ratio, *RT* radiotherapy, *DCIS* ductal carcinoma in situ, *ER* estrogen receptor, *PR* progesterone receptor status.

### Risk of SHMs by radiotherapy

Univariable (Supplementary Table 6) and multivariable Fine-Gray competing risk regression analyses (Table 2) were conducted to assess associations between clinicopathologic factors and risk of SHMs among DCIS survivors. RT was associated with increased risk of SHM (combined as a group) compared with DCIS patients not treated with RT (HR, 1.38; 95% CI, 1.21 to 1.59; *P* < 0.001). This multivariable analysis was adjusted for age at diagnosis, year of diagnosis, race, tumor size, nuclear grade, ER/PR status and surgery. In the analyses of separate SHMs, significantly elevated risks were found for ALL (HR, 3.15; 95% CI, 1.21 to 8.17; *P* = 0.02) and NHL (HR, 1.33; 95% CI, 1.09 to 1.62; *P* < 0.001); marginally significant was CML (HR, 1.69; 95% CI, 0.97 to 3.00; *P* = 0.06) while differences in other SHMs were not significant (Table 2). SIRs adjusted for age, race and year of DCIS diagnosis were computed to compare the incidence of SHMs among survivors of DCIS with the incidence rates of these HMs in the general US population. The results above were computed using the Gray method which controls for competing risks. As a validation of this approach, we also computed standardized incidence ratios (SIR), referred to in Supplementary Note as relative risks, i.e. observed/expected cases. This yielded, for the development of all SHMs combined after RT (SIR, 116; 95% CI, 107 to 126; *P* < 0.001) and (SIR, 95; 95% CI, 89 to 103; *P* = 0.2) for the non-RT group (i.e. not differing from 100 and thus from background rates, as expected). When analyzed by SHM type, SIRs after RT were significantly higher for ALL, CML and NHL (Table 3): SIRs were 380 (95% CI, 163 to 1165, *P* < 0.001) for ALL, 174 (95% CI, 96 to 320, *P* = 0.02) for CML and 120 (95% CI, 102 to 140, *P* = 0.01) for NHL.

**Table 2:** Multivariable competing risk regression analysis of risk of developing hematologic malignancies in subjects with DCIS.

Covariables	ALL		AML		CLL		CML		MM		HL		NHL		SHMs combined	
	HR (95% CI)	*P*	HR (95% CI)	*P*	HR (95% CI)	*P*	HR (95% CI)	*P*	HR (95% CI)	*P*	HR (95% CI)	*P*	HR (95% CI)	*P*	HR (95% CI)	*P*
Age, per year	NS		**1.04 [1.03, 1.05]**	**<0.001**	**1.04 [1.02, 1.05]**	**<0.001**	NS		**1.03 [1.03, 1.04]**	**<0.001**	NS		**1.03 [1.03, 1.04]**	**<0.001**	**1.03 [1.03, 1.04] <0.001**	**<0.001**
Year of diagnosis, per year	**0.92 [0.86, 0.98]**	**0.01**	NS		**0.97 [0.94, 0.99]**	**0.003**	NS		NS		NS		**0.98 [0.97, 0.99]**	**0.001**	**0.98 [0.97, 0.99]**	**<0.001**
Race: Black vs. White	NS		NS		0.86 [0.51, 1.43]	0.56	NS		**1.99 [1.40, 2.83]**	**<0.001**	NS		**0.75 [0.55, 1.02]**	**0.06**	1.02 [0.85, 1.24]	0.92
Race: Others vs. White	NS		NS		**0.16 [0.05, 0.51]**	**0.02**	NS		**1.58 [1.06, 2.35]**	**0.03**	NS		0.94 [0.71, 1.24]	0.64	0.85 [0.69, 1.04]	0.35
Tumor size: 10–19 vs. 1–9 mm	NS		NS		NS		NS		NS		NS		0.89 [0.71, 1.12]	0.33	0.95 [0.81, 1.12]	0.82
Tumor size: 20–49 vs. 1–9 mm	NS		NS		NS		NS		NS		NS		0.80 [0.61, 1.06]	0.12	0.85 [0.70, 1.03]	0.10
Tumor size: 50+ vs. 1–9 mm	NS		NS		NS		NS		NS		NS		0.95 [0.60, 1.51]	0.83	0.98 [0.71, 1.36]	0.83
Grade: II vs. I	NS		NS		NS		NS		NS		NS		NS		0.98 [0.79, 1.21]	0.95
Grade: III vs. I	NS		NS		0.62 [0.36, 1.08]	0.09	NS		NS		NS		NS		1.11 [0.90, 1.37]	0.07
ER: positive vs. negative	0.31 [0.09, 1.12]	0.07	NS		NS		NS		NS		NS		NS		1.00 [0.75, 1.34]	0.31
PR: positive vs. negative	NS		NS		NS		NS		NS		NS		**0.76 [0.58, 0.99]**	**0.04**	0.90 [0.69, 1.16]	0.11
Surgery: mastectomy vs. BCS	0.34 [0.04, 2.66]	0.30	NS		0.80 [0.53, 1.22]	0.30	NS		NS		NS		1.11 [0.86, 1.43]	0.41	1.08 [0.90, 1.29]	0.55
Treatment: RT vs. Non-RT	**3.15 [1.21, 8.17]**	**0.02**	NS		NS		1.69 [0.97, 3.00]	0.06	NS		NS		**1.33 [1.09, 1.62]**	**<0.001**	**1.38 [1.21, 1.59]**	**<0.001**

Shown are HRs and 95% CIs for developing a nonsynchronous (≥1 year after DCIS diagnosis) SHM in patients with DCIS, calculated using Fine-Gray competing risk regression analyses. Covariables that were significant in univariable analyses (*P* < 0.1) were included in the multivariable analysis, which was subjected to the backward selection procedure to generate the final model. The large sample size of the SHMs combined analysis allowed for inclusion of all covariables in the multivariable model, which was also subjected to a backward selection procedure. Univariable regression analyses are shown in the Data Supplement.

Bold type indicates the numbers that remained significant in multivariate analysis.

*ALL* acute lymphocytic leukemia, *AML* acute myeloid leukemia, *CLL* chronic lymphocytic leukemia, *CML* chronic myeloid leukemia, HL Hodgkin lymphoma, *HR* hazard ratio, *MM* multiple myeloma, *NHL* non-Hodgkin lymphoma, *NS* not significant in univariable analysis, *SHM* second hematologic malignancy, *DCIS* ductal carcinoma in situ of the breast.

**Table 3:** SIRs of second hematologic malignancies in patients with DCIS.

SHMs	Non-RT				RT				*P*	Additional risk from RT	
	Observed	Expected	SIR (95% CI)	*P*	Observed	Expected	SIR (95% CI)	Number needed to treat to develop a SHM		SIR (95% CI)	*P*
SHMs combined	727	762	95 (89 to 103)	0.20	562	484	116 (107 to 126)	2360	<0.001	109 (122 to 136)	<0.001
ALL	7	12	57 (23 to 117)	0.15	17	8	209 (122 to 334)	78,003	0.01	380 (163 to 1165)	<0.001
AML	72	78	93 (73 to 117)	0.50	58	49	119 (90 to 154)	22,863	0.20	128 (90 to 181)	0.15
CLL	116	118	98 (81 to 118)	0.85	79	72	109 (87 to 136)	16,785	0.41	111 (83 to 147)	0.40
CML	22	20	108 (68 to 163)	0.65	24	13	188 (121 to 280)	55,252	0.002	174 (96 to 320)	0.02
MM	137	144	95 (80 to 112)	0.56	99	93	106 (86 to 129)	13,394	0.53	112 (86 to 145)	0.39
HL	17	20	84 (49 to 134)	0.50	13	13	98 (52 to 167)	102,004	0.99	117 (53 to 244)	0.50
NHL	356	369	97 (87 to 107)	0.50	272	236	115 (102 to 130)	4875	0.02	120 (102 to 140)	0.01

Not including second malignant neoplasms that occurred in the first year after DCIS diagnosis. An SIR of 100 indicates a similar ratio as the background population.

*DCIS* ductal carcinoma in situ, *ALL* acute lymphocytic leukemia, *AML* acute myeloid leukemia, *CLL* chronic lymphocytic leukemia, *CML* chronic myeloid leukemia, *MM* multiple myeloma, *HL* Hodgkin lymphoma, *NHL* non-Hodgkin lymphoma, *HR* hazard ratio, *RT* radiotherapy, *SHM* second hematologic malignancy, *SIR* standardized incidence ratio.

### Risk dynamics of SHMs after RT

**Fig. 2:**
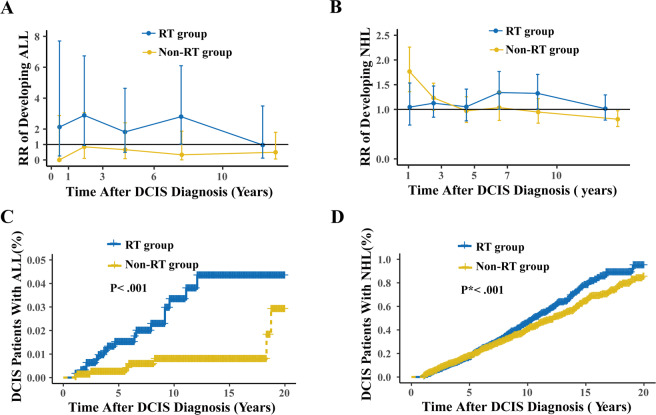
Risk time courses for developing second hematologic malignancy (SHM) after diagnosis of ductal carcinoma in situ (DCIS) of the breast. **a**, **b** Plotted are mean relative risks (RRs)±95% CIs of developing (**a**) acute lymphoblastic leukemia (ALL) and (**b**) non-Hodgkin lymphoma (NHL) as second cancer, on the basis of radiotherapy (RT) compared with the background US population, which is represented by the horizontal black line at y = 1. The number of person-years at risk, expected and observed cases, RRs, and 95% CIs for each RR in time course graph, are shown in the Data Supplement. Risk-time courses for SHMs other than ALL or NHL are also shown in the Data Supplement. **c,** **d** Plotted are the percentage of patients with DCIS diagnosed with (**b**, **f**) AML or (**d**, **h**) CML as function of the years after WDTC diagnosis. In time-to-event analyses, only patients that have ≥1 year of follow-up after DCIS diagnosis are included (164,540 DCIS patients in total). Patients were censored at death, if still alive on December 31, 2016, or when they developed a non-SHM second cancer. Additional hazard curves are shown in the Data Supplement.

RR time courses and time-to-event courses of SHMs development in DCIS patients are shown in Fig. 2 and Supplementary Fig. 3/ Tables 7–14. Compared with the background incidence rate of ALL, we observed persistently increased risks of ALL (Fig. 2a and Supplementary Table 8) in the first 10 years after RT among patients with DCIS that peaked in the second year (RR, 2.89; 95% CI, 0.94 to 6.74; *P* = 0.05) and eighth year (RR, 2.80; 95% CI, 1.03 to 6.10; *P* = 0.03). The risk of ALL declined and reached baseline rates within 10 years after DCIS diagnosis. Similar risk dynamics were found for CML, but with RRs for RT vs no RT differing only marginally (Supplementary Fig. 3, Supplementary Table 11). As for NHL, we observed a delayed increase in the risk of NHL in patients with DCIS treated with RT that was sustained 5–10 year after treatment (RR for years 5–7, 1.34; 95% CI, 1.00 to 1.77; *P* = 0.04; RR for years 7–10, 1.33; 95% CI, 1.01 to 1.71; *P* = 0.03; Fig. 2c and Supplementary Table 14). In time-to-event analysis, RT was associated with ALL (absolute10-year occurrence rate difference, 0.03%; *P* < 0.001; Fig. 2b) and NHL (absolute10-year occurrence rate difference, 0.1%; *P* < 0.001; Fig. 2D) risk compared with non-RT group, whereas no significant association was observed in other SHMs (Supplementary Fig. 3/ Tables 7–14).

### Risk of SHMs in low-/intermediate-risk DCIS

When examining risks of SHMs from RT in low-/intermediate-risk DCIS (Supplementary Tables 15–19), where RT carries no or questionable clinical benefit^8^ because risk of local recurrence is minimal, and as such, individualized decision-making is suggested^13^, we found, stratifying by age at diagnosis, tumor size and grade, that RT was associated with increased risk of ALL (cases only occurred in RT group; No. cases: 5/100,000 person-years) and NHL (HR, 1.32; 95% CI, 1.00 to 1.73, *P* = 0.048, No. cases: 63/100,000 person-years) in multivariable competing risk regression analysis (Supplementary Tables 20 and 21).

### Outcomes after development of ALL and NHL

**Fig. 3:**
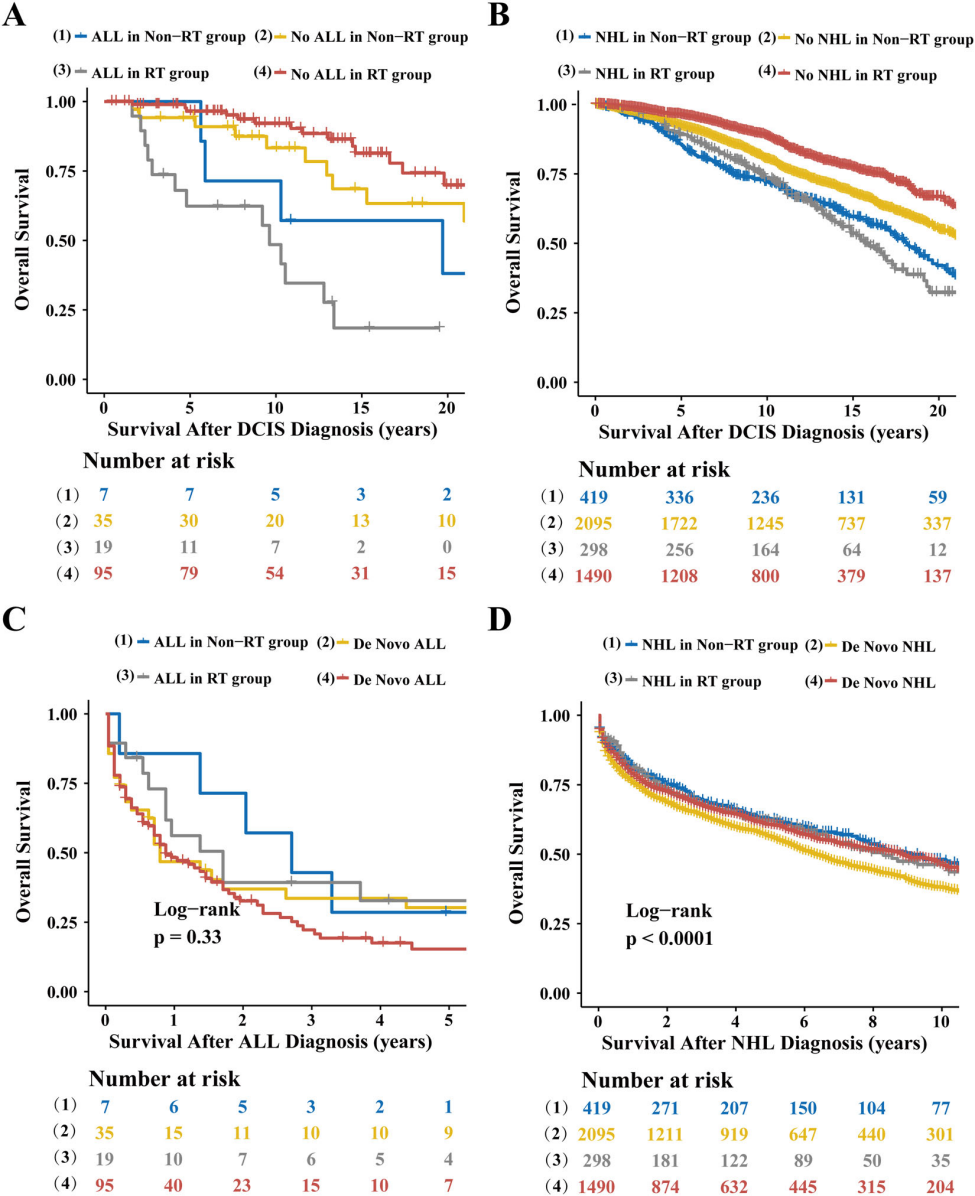
Survival curves of patients with ductal carcinoma in situ (DCIS) of the breast by development of acute lymphoblastic leukemia (ALL) or non-Hodgkin lymphoma (NHL) and by radiotherapy (RT). **a–d** Shown are Kaplan**–**Meier plots of case-control studies wherein the following groups were compared: patients with DCIS who developed **a** ALL or **b** NHL after DCIS diagnosis (cases) versus those who did not (controls); **c** patients with ALL and **d** NHL who were diagnosed with these diseases after DCIS diagnosis (cases) versus those who developed ALL or NHL de novo (controls). In all figures, (2) are matched controls for (1), and (4) are matched controls for (2). P values were calculated using the log-rank test. *P values were calculated using a two-stage procedure test.

As expected, DCIS patients who developed ALL or NHL (Supplementary Tables 22–26) had shorter OS than matched subjects who did not develop any SHMs (median OS for ALL, 10.3 years vs. 31.3 years; *P* < 0.001; Fig. 3a; median OS for NHL, 17.4 years vs. 24.5 years; *P* < 0.001; Fig. 3b), regardless of whether RT was given or not. Among DCIS patients who developed NHL, RT was associated with worse OS compared to the non-RT group (median OS, 15.7 years vs. 18.1 years; *P* < 0.001, Fig. 3b). To compare the OS difference between patients with de novo hematologic malignancy and SHM, we defined OS as the time between SHM diagnosis and death from any cause. There was no significant difference in survival between patients with de novo ALL and those who developed ALL in the RT group (median OS, 2.7 years vs. 0.8 years; *P* = 0.54) or non-RT group (median OS, 1.7 years vs. 0.9 years; *P* = 0.18, Fig. 3c). Compared with matched controls with de novo NHL, DCIS patients that developed NHL had better OS in non-RT group (median 9.1 years vs. 6.3 years; *P* < 0.001), but similar OS in RT group (median 8.3 years vs. 8.9 years; *P* = 0.73, Fig. 3d).

## Discussion

Given DCIS incidence is rising rapidly due to increased screening^1^, long-term survivors of DCIS have a greater cumulative probability of experiencing SHMs induced by therapies^16,17^. As SHMs are generally refractory to treatment and associated with poor prognosis^31–34^, excess use of RT, especially in low-risk cases, requires greater scrutiny to better inform DCIS patients and clinicians of risks vs benefits. To comprehensively describe this potentially lethal risk, we evaluated 184,363 patients diagnosed with DCIS over four decades. Our study indicates that compared to background incidence rates in the US, DCIS patients receiving RT have significantly increased risks of ALL, CML, and NHL; elevated risks of ALL, NHL and marginally CML, were also observed among low-/intermediate-risk patients with DCIS when compared to counterparts without RT; there is no obvious latency period for ALL and CML, risks of which elevate for 10 years after RT and decline to baseline thereafter, while for NHL a 5-year latency and a peak at 5–10 years after DCIS diagnosis was shown; shorter survival in DCIS patients who developed ALL, which was not influenced by receipt of RT; and development of NHL in DCIS patients with RT presenting worse survival than counterparts without RT.

Generally speaking, most secondary malignancies arising from a course of RT are in organs contiguous with radiation target, such as secondary lung cancer, thyroid cancer, esophageal cancer, and melanoma of the skin among breast cancer survivors^17,35^, so physicians are likely to ignore SHM. An intriguing, and clinically relevant finding of our study was that we identified 21(1.6%) SMHs located in the breast, the vast majority of which were NHL (20/21) and in the RT group (18/21). Of note, diffuse large cell lymphoma, a type of extra-nodal NHL, is a rare disease but associated with breast implants^36,37^, which was also found among most NHL located in the breast (18/20) in this study. Biologically, although radiation for DCIS includes only partial rib irradiation (in contrast to regional node irradiation in cases of invasive breast cancer which includes part of the sternum), RT associated exposures to high acute doses of ionizing radiation causes somatic mutations and chromosomal alterations that may also lead to leukemia^38^ and other myeloid malignancies^39,40^. This increased risk of RT adds another dimension to the question of whether DCIS patients should choose mastectomy as opposed to BCS^22^.

Our results indicate that exposure to external beam RT was associated with persistent risk of ALL and CML within 10 years after DCIS diagnosis. It is well known that ALL occurs in both children and adults but its incidence peaks between 2 and 5 years of age^41^ (Supplementary Fig. 2). We still observed a small number of developments of ALL in this cohort with a median age of 58 years, but considerable magnitudes of relative risk between RT, Non-RT groups relative to background. Prior molecular studies suggested that ionizing radiation may affect NHL risk either indirectly through long-term immunosuppression^42^ or directly by DNA damage^43^ in the lymphocytes, which are well-established risk factors for NHL^44,45^. Interestingly, the patterns of incidence and latency of secondary lymphomas is distinct from that of other HM or solid malignancies^25^. While many scholars hold the view that NHL risk associated with radiotherapy is expected to occur 5 or more years after exposure^46–50^, our study is the first to confirm this delayed risk in DCIS patients.

Expectedly, patients with DCIS who developed ALL or NHL had an inferior OS compared with those who did not develop ALL or NHL, which is supported by the fact that occurrence of second cancers in first unrelated primary cancer survivors increases mortality^27,51^. Moreover, we observed that the detrimental impact of RT on survival is substantial in DCIS patients developing NHL, where DCIS patients with second NHL in the RT group had a worse survival than those in the Non-RT group, and findings from a previous study^28^ were consistent with that. Comparable OS was observed in radiation-exposed second NHL subjects and de novo NHL patients. Of note, the median age of DCIS patients developing NHL is 65 years. This is consistent with prior observations that the impact of second primary malignant neoplasms on survival was more pronounced in young adults than older adults, where adolescents and young adults with second malignancies had a greater than 2-fold increased risk of cancer-specific death relative to those with the same de novo neoplasms^27^.

An intriguing and clinically relevant finding of our study was that among low-/intermediate-risk DCIS patients defined by Smith et al.^9^ whose patient prognostic score ranged from 0 to 2, an increased risk of ALL and NHL was observed in the RT group compared to the non-RT group. Prior studies indicated that DCIS patients with combined low-risk characteristics may be adequately treated with breast conserving surgery alone, the majority of them with no benefit from RT^8,12^. RT decisions are multifactorial, with SHM risk being one in a large number of individualized considerations for the use of RT due to its low absolute risk given in this study (105 cases per 100,000 person-years in RT group). American Society for Radiation Oncology (ASTRO) evidence-based guidelines recommends that a tumor bed boost may be used for patients with DCIS who meet any of the following criteria: less than 50 years, high nuclear grade, or close (<2 mm) or positive margins^13^. While we did not show any dose dependent pattern for SHM risk from RT^52,53^, this risk should be a consideration in RT dose/fraction and the use of boost.

Additionally, it should be recognized that our study cohort spans over 4 decades, where treated fields and techniques used for the delivery of RT have changed^20,54^. Interestingly, when the analysis was confined to the years 2001 to 2016, RT was associated with the increased risk of CML (Supplementary Table 27), while during the period 1975–2000 an increased risk of ALL and NHL was observed with RT (Supplementary Table 28). Older radiation delivery methods, larger treatment fields and inclusion of regional nodal irradiation during the first years of the study period may explain these results^55,56^.

To our knowledge, this is the first comprehensive report of risk dynamics of individual SHM entities over time after RT treatment of DCIS. The large population-based nature of this study offers sufficient patients numbers and long enough follow-up to allow us to detect this rare devastating complication, especially for long latencies to NHL^57^. As patients with DCIS have long disease-free survival periods at risk of competing nonhematologic death risks, we employed Fine-Gray competing risk regressions^16–18^. Furthermore, we employed SEERaBomb^19^, a package in the statistical programming language R^58^, to conduct second cancer risk analyses rather than SEER*Stat, which was developed by the NCI, which accesses only registries in SEER 9 (1975–2016), SEER 13 excluding Alaska (1992–2016), or SEER 18 excluding Alaska (2000–2016) but not all 18 registries from 1975 to 2016, which limits power available to estimate lower intensity risks. Thus, we identified more SHM cases (Supplementary Fig. 1) and a greater proportion of DCIS patients without RT compared to a previous study^59^.

Several limitations of the present study need to be considered. First, margin width, a crucial covariable used to support RT decisions, is not included in SEER data^10,11^. Similarly, SEER data does not include information on endocrine therapy^60,61^, though it is used as an adjuvant treatment after surgery with/without RT for DCIS^62–64^. We also observed that ER/PR-positive tumors treated with RT, had reduced risks of SHMs compared to counterparts with ER/PR-negative diseases. Given the association between RT and endocrine therapy/ER status, the borderline increased risk of NHL associated with radiotherapy may be a spurious result of incomplete adjustment for endocrine therapy. Second, although we only selected patients who received external beam radiotherapy, this database does not include information on treatments fields or radiation doses, and as such, SHM risk in a dose-response manner could not be assessed^52,53,65^. In addition, only initial treatment data are recorded in SEER^66^, so RT administrated for recurrent disease or as a delayed treatment, is not recorded in SEER. Lastly, DCIS patients are relatively old and may have other chronic diseases, so there is a possibility that exposures occur in a variety of other ways, including diagnostic CT scans and accumulative environmental exposure to γ-radiation. As no data are available on these other sources of radiation exposures, we were not able to estimate the direction and magnitude of their influence on our obtained results.

In conclusion, this research provides timely evidence that DCIS patients receiving RT have an elevated risk of developing ALL and NHL. Our findings highlight the importance of avoiding or reducing RT when treating low/intermediate-risk disease receiving breast conserving surgery and at least minimizing RT in high-risk cases. Finally, long NHL latencies demand continuous monitoring of SHM in DCIS survivors.

## Methods

### Study design and participants source

This longitudinal cohort study used the April 2019 release of the Surveillance, Epidemiology, and End Results (SEER) database of the National Cancer Institute (NCI). This database includes 18 population-based cancer registries covering 34.6% of the US population^67^. Patients were excluded from our analysis if: the breast malignancy was not DCIS histologically (Supplementary Note), e.g., if it was Paget’s disease or DCIS with micro-invasion; if DCIS was not the person’s first cancer; if the hematologic malignancy (HM) was a first, third, or higher order primary cancer; if chemotherapy was received; and if RT or survival status was unknown (Fig. 1). In total, we included 184,363 eligible DCIS patients: 77,927 in RT group and 106,436 in non-RT group. HM in this study included acute lymphoblastic leukemia (ALL), acute myeloid leukemia (AML), chronic lymphocytic leukemia (CLL), chronic myeloid leukemia (CML), multiple myeloma (MM), Hodgkin lymphoma (HL) and non-Hodgkin lymphoma (NHL), which were defined according to International Classification of Diseases for Oncology (3rd edition) (ICD-O-3) histology codes and ICD-9/10 codes (Data Supplement). Low-/intermediate-risk patients with DCIS were defined as having overall patient prognostic scores of 0 to 2 according to Van Nuys Prognostic Index (VNPI)^9,68,69^. Informed consent was obtained from all patients included in the SEER study. Given that the SEER program provides de-identified information of patients, the Chongqing Medical University Institutional Review Board considers SEER data analyses to be exempt from Institutional Review Board review.

### Procedures

The R package SEERaBomb^19^ was used to query all 18 SEER registries to identify patients diagnosed with a primary DCIS who were treated with or without RT and developed SHM. An advantage of SEERaBomb over SEER*Stat MP-SIR (Multiple Primary-Standardized Incidence Ratio), developed by the NCI, is that it uses more of the data and thus allows higher resolution second cancer risk estimates (Supplementary Fig. 1). This allows greater resolution of SHM risk dynamics after diagnosis of first cancers^19^. Relative risk (RR) time courses for developing SHM after DCIS treatment were computed as ratios of observed and expected SHM cases for each treatment group. Herein, the expected number of patients with SHM for DCIS cases with or without receiving RT was calculated using age-sex-year specific background incidence rates of HMs in the general US population^70^ and multiplying them by corresponding age-sex-year specific person-years (PYs) at risk for a SHM after DCIS diagnosis, summing over all patients and all ages and years.

### Outcomes

The primary outcome was the development of SHM, defined as a nonsynchronous SHM occurring ≥1 year after DCIS diagnosis^71^. In addition, we conducted survival analyses using two separate case-control designs to assess overall survival (OS) of patients with DCIS who developed an SHM, where each patient with DCIS who developed SHM was compared with either five patients with DCIS who did not develop SHM or with five de novo HM patients. Propensity score matching was used to balance clinicopathological characteristics of DCIS/SHM between groups^72^, where we calculated propensity scores based on age at diagnosis, year of diagnosis, race, tumor size, nuclear grade, estrogen receptor (ER)/progesterone receptor (PR) status and surgery types, using logistic regressions.

### Statistical analysis

Demographic and clinicopathological characteristics of cases are presented using medians (interquartile ranges [IQR]) for continuous variables not normally distributed (as indicated by Kolmogorov-Smirnov normality test, *P* < 0.05) and frequencies (and percentages) for categorical variables. Mann–Whitney U, Pearson Chi-Square, and Fisher’s exact tests were employed to test differences in medians and proportions of continuous and categorical variables between the RT and non-RT group. The calculation of RR and RT-attributable RR ratios with 95% confidence interval (CIs) and *P*-values is described in the Data Supplement^73^. Given the low occurrence rate of SHM and long-term follow up, we employed Fine-Gray competing risk regression analyses to estimate sub-distribution hazard ratios (HRs) and 95% CIs^74^, where competing events included secondary malignancies other than HMs and death from any cause. Time-to-SHM-event was defined as date from DCIS diagnosis until SHM diagnosis, and censoring time for SHM incidence was defined as date from DCIS diagnosis until development of SHMs other than the HM of interest, death, last contact (through December 31, 2016), or 20 years after DCIS diagnosis, whichever occurred first. Variables were included in the multivariate proportional hazard regression full model if P values (for differing from zero) were less than 0.1 (two-sided) in univariate analyses; Gray’s competing risk R package cmprisk was used for this. Final multivariable models were determined from the full model by applying backward model selection [using step() in R]. Relative risks for SHMs vs the general US population were also determined by estimating age-sex-year specific SIRs. The Kaplan-Meier method was used to estimate plotted survival probabilities. *P*-values for differences between OS curves were calculated using the log-rank test, with one exception: the two-stage procedure test was used when survival curves crossed each other^75,76^. All analyses were conducted using R version 3.6.2; scripts used are provided in the Data Supplement.

### Reporting summary

Further information on research design is available in the Nature Research Reporting Summary linked to this article.

## Supplementary information

Data Supplements

Reporting Summary Checklist

## Data Availability

The data generated and analyzed during this study are described in the following data record: 10.6084/m9.figshare.13547411^77^. Data analyzed during the study are openly available via the National Cancer Institute’s Surveillance, Epidemiology, and End Results program (SEER) (https://seer.cancer.gov/). The data generated in the study are contained in the file ‘cancDef.Rdata’. This file was derived from the authors’ GitHub package “SEERaBomb” (https://github.com/radivot/SEERaBomb). Details of how to use the package to acquire the data are provided with the GitHub package.

## Code availability

The code developed during this study is available upon reasonable request (https://github.com/KangWang0822/SHM-risk-among-DCIS-survivors).
