# Supplementary material for: Risk of hematologic malignancies after breast ductal carcinoma in situ treatment with ionizing radiation
Source: NPJ Breast Cancer. 2021 Mar 2;7:21. doi: 10.1038/s41523-021-00228-6 (PMC7925676; doi:10.1038/s41523-021-00228-6)
Supplement: Supplementary file 1 — Data Supplements [file 41523_2021_228_MOESM1_ESM.pdf]

ONLINE DATA SUPLEMENT TO

Risk of Hematologic Malignancies after Breast Ductal Carcinoma in Situ Treatment with Ionizing Radiation

BY Kang Wang ET AL

Npj Breast Cancer

Contents

Supplementary Note **4**

Background Information on SEER Databases 4

Comparison Between SEERaBomb and SEER*Stat 4

Acquisition of SEER Data 4

Inclusion of Hematological Malignancy Subtypes 4

Procedures to Estimate Relative Risk Dynamics 5

Procedures to Estimate RT-Attributable Relative Risk Ratios 6

Procedures to Estimate Standardized Incidence Ratios 6

Additional Information on Covariates Extracted From SEER 6

Fine and Gray Competing Risk Regression Analyses to Calculate Hazard Ratios of RT to Develop SHM 7

Supplementary Figures **8**

Supplementary Figure 1: Comparison of SEER*Stat and SEERaBomb Coverage for Second Cancer Analyses. 8

Supplementary Figure 2: Age-Versus-Risk Plots of SHM Incidence De Novo and After DCIS Diagnosis. 9

Supplementary Figure 3: Risk-Time Courses of SHMs After DCIS Diagnosis 11

Supplementary Figure 4: Risk-Time Courses of SHMs After Low/intermediate-risk DCIS Diagnosis. 13

Supplementary Figure 5: Risk-Time Courses of SHMs After DCIS Treatment by Age. ... 14

Supplementary Figure 6: Time-to-Event Curves of SHM Development After Treatment for DCIS by Age. 15

Supplementary Tables 16

Supplementary Table 1: ICD Codes of Included ductal carcinoma in situ of the breast and Hematological Malignancies. 16

Supplementary Table 2: All Hematologic and Non-Hematologic Second Cancers Identified After DCIS Diagnoses. 19

Supplementary Table 3: Baseline characteristics of DCIS patients who later developed ALL. 20

Supplementary Table 4: Baseline characteristics of DCIS patients who later developed CML. 22

Supplementary Table 5: Baseline characteristics of DCIS patients who later developed NHL. 24

Supplementary Table 6. Univariable Competing Risk Regression Analysis of Risk of Developing Hematologic Malignancies in subjects with DCIS. 26

Supplementary Table 7: Relative Risk-Time Course Specifics for Each Time Interval: Any SHM After DCIS dignosis. 27

Supplementary Table 8: Relative Risk-Time Course Specifics for Each Time Interval: ALL After DCIS dignosis. 27

Supplementary Table 9: Relative Risk-Time Course Specifics for Each Time Interval: AML After DCIS dignosis. 27

Supplementary Table 10: Relative Risk-Time Course Specifics for Each Time Interval: CLL After DCIS dignosis. 28

Supplementary Table 11: Relative Risk-Time Course Specifics for Each Time Interval: CML After DCIS dignosis. 28

Supplementary Table 12: Relative Risk-Time Course Specifics for Each Time Interval: MM After DCIS dignosis. 28

Supplementary Table 13: Relative Risk-Time Course Specifics for Each Time Interval: HL After DCIS dignosis. 29

Supplementary Table 14: Relative Risk-Time Course Specifics for Each Time Interval: NHL After DCIS dignosis. 29

Supplementary Table 15: Relative Risk-Time Course Specifics for Each Time Interval: ALL After Low/Intermediate-Risk DCIS dignosis. 29

Supplementary Table 16: Relative Risk-Time Course Specifics for Each Time Interval: CML After Low/Intermediate-Risk DCIS dignosis. 30

Supplementary Table 17: Relative Risk-Time Course Specifics for Each Time Interval: NHL After Low/Intermediate-Risk DCIS dignosis. 30

Supplementary Table 20. Univariable Competing Risk Regression Analysis of Risk of Developing Hematologic Malignancies in subjects with Low/Intermediate-Risk DCIS. ... 33

Supplementary Table 21. Multivariable Competing Risk Regression Analysis of Risk of Developing Hematologic Malignancies in subjects with Low/Intermediate-Risk DCIS. ... 34

Supplementary Table 22: Characteristics of DCIS Cases and Controls. 35

Supplementary Table 23: Characteristics of ALL, CML and NHL Cases and Controls..... 37

Supplementary Table 24: Histologic Characteristics of ALL Cases and Controls............. 38

Supplementary Table 25: Histologic Characteristics of CML Cases and Controls............ 38

Supplementary Table 26: Histologic Characteristics of NHL Cases and Controls. 38

Supplementary Table 27. SIRs of Second Hematologic Malignancies in Patients With DCIS diagnosed from 2001 to 2016. 40

Supplementary Table 28. SIRs of Second Hematologic Malignancies in Patients With DCIS diagnosed from 1975 to 2000. 41

Supplemental References 42

Supplementary Note

Background Information on SEER Databases

The Surveillance, Epidemiology, and End Results (SEER) database (http://seer.cancer.gov/)[1], sponsored by the US National Cancer Institute, began in 1973 with 9 registries and the number of registries increased from 9 to 13 (SEER 13) in 1992 and from 13 to 18 in 2000 (SEER 18). The cancer data of SEER 18 represents a large proportion of Americans, including 26 percent of African Americans, 38 percent of Hispanics, 44 percent of American Indians and Alaska Natives, 50 percent of Asians, and 67 percent of Hawaiian/Pacific Islanders. The SEER9 starting from 1973 contains more person-years (PY) of risk than others (SEER13 and SEER18), also, it had greatest number of cancer cases. We employed the SEERaBomb[2], a program for the statistical programming language R[3] to conduct second cancer risk analyses rather than SEER*Stat MP-SIR (Multiple Primary-Standardized Incidence Ratio), a statistical tool made publicly available by SEER, where we had previously validated its inherent limitations.

Comparison Between SEERaBomb and SEER*Stat

The SEER*Stat MP-SIR can access only registries in SEER 9 (1973-2016) or SEER 13 excluding Alaska (1992-2016) or SEER 18 excluding Alaska (2000-2016) but not all 18 registries from 1973 to 2016, and theoretically lead to underestimation of the population-at-risk. Although SEER 18 could access all the cancer patients diagnosed after 2000, except for cases in Alaska, this setting results in eliminating some secondary cases whose prior cancers were diagnosed before 2000. Therefore, we should make comparison of SEER*Stat and SEERaBomb coverage for second hematological malignancies (SHM) analyses (Supplementary Figure 1).

Acquisition of SEER Data

ASCII text data is an optimum data input for SEERaBomb, downloaded from https://seer.cancer.gov/data/options.html after completing and submitting a SEER Research Data Agreement as well as a NCI-SEER Custom Data Agreement (available via seercustomdata@imsweb.com), which can access radiotherapy/chemotherapy data. Once ASCII file was acquired, the SEERabomb was used to transform it to a R-datafame that was analyzed for secondary cancer risk. To install SEERaBomb, the instruction contained in the website (http://epbi-radivot.cwru.edu/SEERaBomb/SEERaBomb.html) could be referred. In addition, some detailed functions of SEERaBomb were introduced in github (https://github.com/radivot/SEERaBomb).

Inclusion of Hematological Malignancy Subtypes

SHM involved in this study were Acute myeloid leukemia (AML), chronic myeloid leukemia (CML), acute lymphoblastic leukemia (ALL), chronic lymphocytic leukemia (CLL), Hodgkin lymphoma (HL),

non-Hodgkin lymphoma (NHL) and multiple myeloma (MM), which were defined by the International Classification of Diseases for Oncology, Third Revision (ICD-O-3) histology codes and ICD-9/10 codes (Supplementary Table 1). Myelodysplastic syndromes (MDS) and Philadelphia chromosome-negative myeloproliferative neoplasms (Ph- MPN) were excluded in this study due to following uncertain issues: (1) undercapturing of MDS and Ph- MPN cases in SEER and inherent small sample sizes, diagnostic uncertainties (histopathological confirmation being not an essential criteria for diagnostic confirmation and reporting); (2) inability to separate de novo AML from AML arising from MDS or Ph- MPN (SEER has been reporting progression of MDS or Ph- MPN to AML only since 2010); (3) short study period from 2001 to 2016 (compared to 1973 to 2016 for DCIS and other SHM).

Procedures to Estimate Relative Risk Dynamics

SHM second cancer risk dynamics after diagnosis of DCIS treated with breast conserving surgery (BCS) and BCS + radiotherapy (RT) were estimated using the same methodology as published earlier and summarized below. We fitted the following generalized additive model to cases observed using Poisson regression[4]: cases ~ s(age) + s(year) + ti(age, year) + offset(log(PY)) to estimate the expected number of SHM if risks are at background rates. Population PY in the age group 85+ years were given as a single value and were therefore redistributed to ages 85 to 99 years using male and female US National Vital Statistic Report mortality rates of 2001 (URL: https://www.cdc.gov/nchs/products/nvsr.htm). In SEERaBomb, when a SEER subject is diagnosed with a first cancer, such as DCIS, the patient's PYs at risk for an SHM becomes a strip of time that is diagonally directed across ages and calendar years in a single-year resolution PY matrix that has years as columns and ages as rows. The orientation of the strip is diagonal because each increase of one year of age implies a proportional increase of one year of calendar time. For each SEER cancer patient, PY strips add values between 0 and 1 to matrix elements under the strip. Fractions are calculated based on months of survival. Resolution of ages was prioritized over calendar years as typically, the correlation between second cancer incidence and age is stronger than the association between second cancer incidence and calendar year of diagnosis. Such PY matrices were generated for each selected time interval after diagnosis of DCIS. PY strip start and end ages were calculated as DCIS age-at-diagnoses plus starting and ending times of the time-since-diagnosis interval of interest, clipped by age-at-diagnosis of an SHM and survival times, whichever came first. In other words, DCIS patients were censored at their month of death when they did not develop an SHM after their DCIS diagnosis. Because of computational efficiency, PY strips were summed using C++ (via the package Rcpp, for the R statistical programming language) and all other codes were written in R. Background incidences that were multiplied point-wise into PY matrices of specific time-since-diagnosis intervals were summed over product matrix elements to form expected numbers of SHM cases after DCIS diagnoses for a specific time interval (E) which could be compared to the observed numbers of SHM cases after DCIS diagnosis for the corresponding time interval (O). This yielded relative risks (RR) = O/E. RR 95% confidence intervals (CI) were calculated in R under the assumption that O is Poisson distributed as qchisq(0.025, 2*O)/(2*E) and qchisq(0.975, 2*O+2)/(2*E). In summary, we determined O and calculated E for predefined time intervals [0,1), [1,3), [3,6), [6,10) and [10,end) years after DCIS diagnosis and from those, RRs and 95% CIs were calculated. Time courses of RRs ± 95% CIs for the risk of developing SHM were then plotted at interval midpoints.

Procedures to Estimate RT-Attributable Relative Risk Ratios

RT-attributable relative risk ratios (RRRRs) were calculated as RR_{RT}/RR_{BCS}, which can also be written as $O_{RT}/O_{Non\text{-}RT} \times E_{RT}/E_{Non\text{-}RT}$ 95% CIs for RRRRs were 2.5 and 97.5% quantiles of 5000 simulations of $(O_{RT}/O_{Non\text{-}RT}) \times (E_{RT}/E_{Non\text{-}RT})$ with O_{RT} and $O_{Non\text{-}RT}$ Poisson distributed with means equal to observed values with and without RT for DCIS. For RRRR figures, we calculated O and E for predefined time intervals [0,1), [1,3), [3,6), [6,10) and [10, end) after DCIS diagnosis and from those, RRRRs and 95% CIs were calculated.

Procedures to Estimate Standardized Incidence Ratios

Standardized incidence ratios (SIRs) with 95% CIs shown in Table 3 of the main article text are RRs with 95% CIs calculated as described in the previous paragraph for the time interval [1,20) years after DCIS diagnosis. RT-attributable SIRs with 95% CIs are RRRRs with 95% CIs calculated as described in the previous paragraph for the time interval [1,20) years after DCIS diagnosis. Follow-up was cut off at 20 years after DCIS diagnosis because SHMs occurring relatively quickly after DCIS treatment may have treatment implications, especially because those SHMs occur in young patients where they have the most devastating impact on patient outcomes.

Additional Information on Covariates Extracted From SEER

(1) Ethnicity/race in SEER is listed as either white, black or other, where "other" includes Asian, American Indian, Alaskan Native, and Pacific Islander ethnicities.
(2) Breast conserving surgery (BCS) were ascertained by 2 SEER columns, Site specific surgery code 10 and 20, which was tracked for cases diagnosed from 1973 to 1997, as well as RX Summ--Surg Prim Site (Surgery of Primary Site) code 20-24 tracked from 1998 through 2016.
(3) DCIS stage was assessed by 2 SEER columns, EOD (EOD stands for "extent of disease") code 0, which was tracked from 1973 through 2003, and CS (Derived from Collaborative Stage) code 0 for cases diagnosed in 2004 and later. The histology of DCIS was ascertained by ICD-O-3 Histology Code (showing details in Supplementary Table 1).
(4) DCIS tumor size is based on 5 SEER columns, EOD-OLD 4 DIGIT (EOD-4), which was tracked from 1983 through 1987, EOD-TUMOR SIZE (also called EOD-10, which was tracked from 1988 through 2003, and CS TUMOR SIZE (CS stands for "collaborative stage"), which was tracked for cases diagnosed in 2004 and later. Older tumor size columns (EOD-2), which were tracked from 1973 through 1982, do not contain objective tumor size descriptives for DCIS, precluding these cases from tumor size analyses. Although we labeled the tumor sizes of these cases as "unknown", we used analyses these cases in low/intermediated risk subgroup based on their scope of tumor size (i.e. <5mm; 5-9mm; 0-19mm; 20-29mm).
(5) DCIS with paget disease or microinvasion were inferred according to 4 SEER columns, EOD-2 code 4, which was tracked from 1973 through 1982, EOD-4 code 5, which was tracked from 1983 through

1987, EOD-10 code 05,20, which was tracked from 1988 through 2003, and CS Tumor Size Code 990, 997, which was tracked for cases diagnosed in 2004 and later.

Fine and Gray Competing Risk Regression Analyses to Calculate Hazard Ratios of RT to Develop SHM

Fine and Gray competing risk regression analyses were the preferred method to calculate hazard ratios (HRs) of developing an SHM after treatment for DCIS because of the low event rate of SHMs under these circumstances. These analyses were performed using the "cmprsk" package and the crr-addson R script described elsewhere[5]. In these regression analyses, SHM was the time-dependent endpoint and death from all causes or development of non-SHM malignancy were treated as competing events to calculate HRs with 95% CIs of developing a SHM after DCIS. Censoring occurred at follow-up cut-off of the April 2019 SEER release (November 2018 submission to the SEER registries), death, development of a second cancer other than the HM of interest or when 20 years of follow-up were reached, whichever occurred first. Univariate and multivariate competing risk regression analyses were respectively performed to calculate HRs for developing SHM after DCIS. Covariates that were significant in univariate analyses ($P < 0.1$) were included in the multivariate analysis, which was subjected to the backwards Wald procedure to generate the final model. Kaplan-Meier method was used to plot the survival curve, whose P value for OS comparison was calculated based on the log-rank test or the two-stage procedure test especially when two survival curves cross each other[6,7].

Supplementary Figures

Supplementary Figure 1: Comparison of SEER*Stat and SEERaBomb Coverage for Second Cancer Analyses.

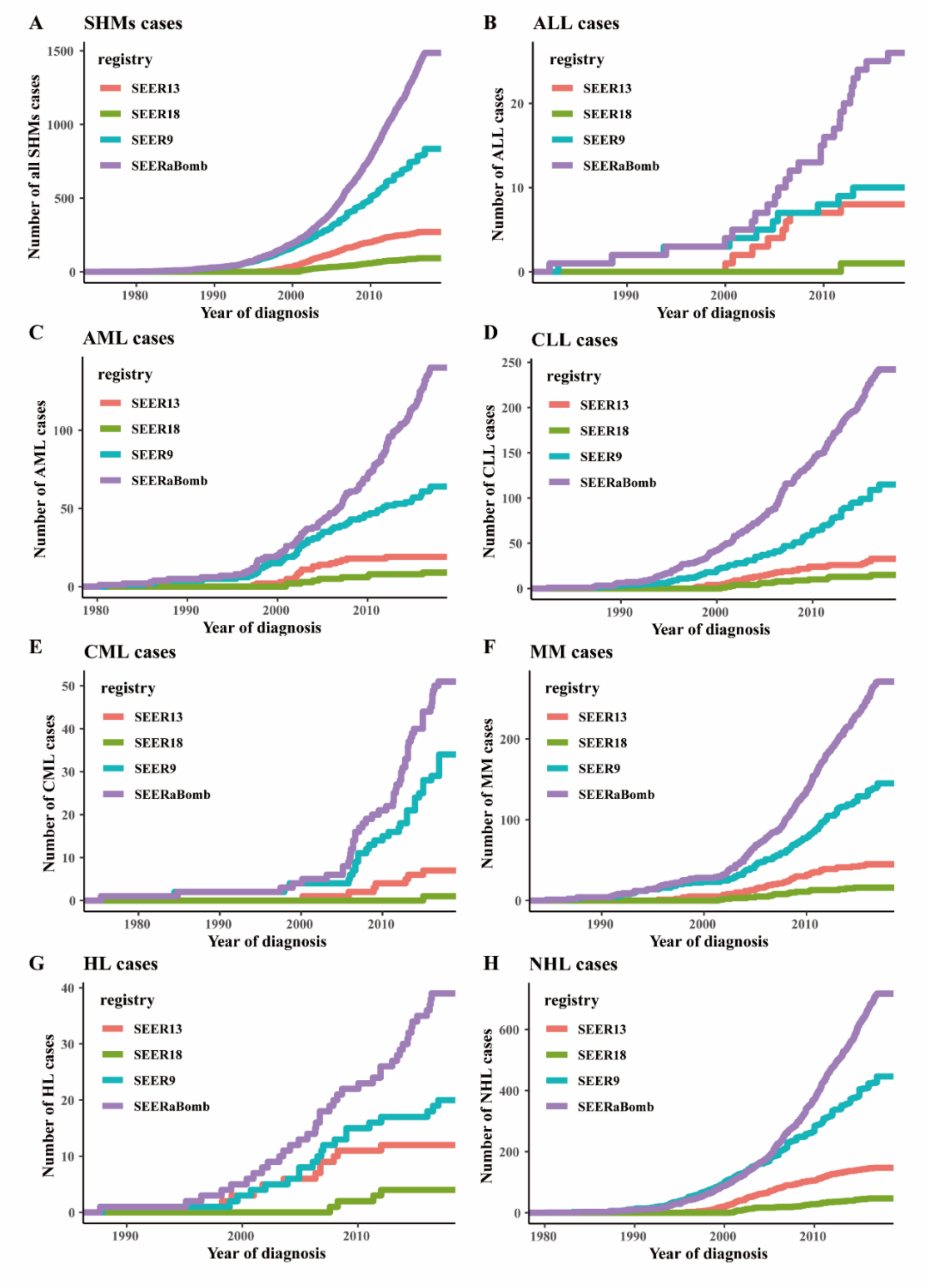

Plotted is the capture of (A) all SHM cases, (B) ALL cases, (C) AML cases, (D) CLL cases, (E) CML cases, (F) MM cases, (G) HL cases and (H) and NHL cases as second cancer after DCIS in either SEERaBomb or the three possible settings of SEER*Stat.

Supplementary Figure 2: Age-Versus-Risk Plots of SHM Incidence De Novo and After DCIS Diagnosis.

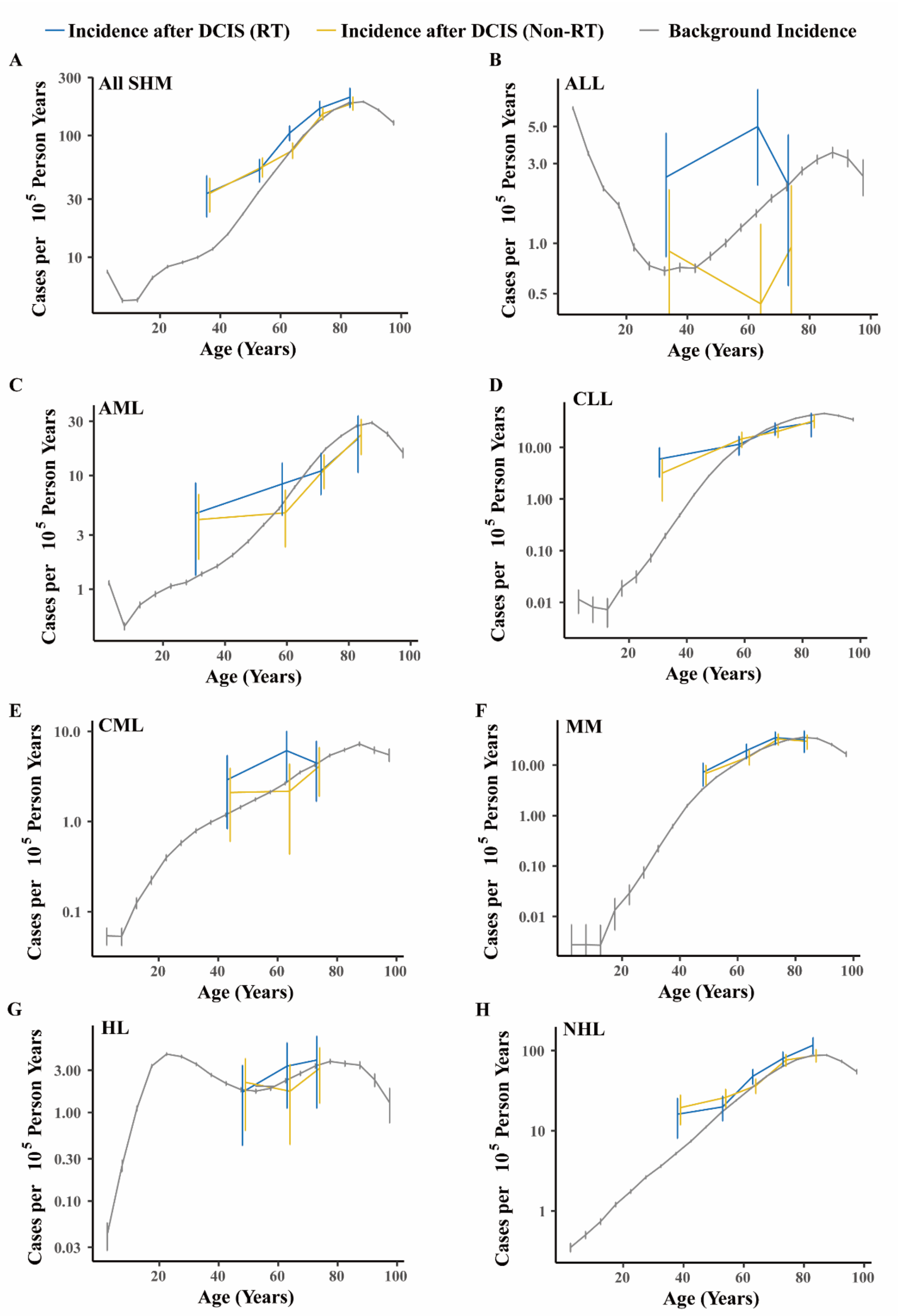

Plotted is the incidence of (A) all SHM cases, (B) ALL cases, (C) AML cases, (D) CLL cases, (E) CML cases, (F) MM cases, (G) HL cases and (H) and NHL cases in the background US population and as

second cancer after DCIS first cancers, per age group.

Supplementary Figure 3: Risk-Time Courses of SHMs After DCIS Diagnosis.

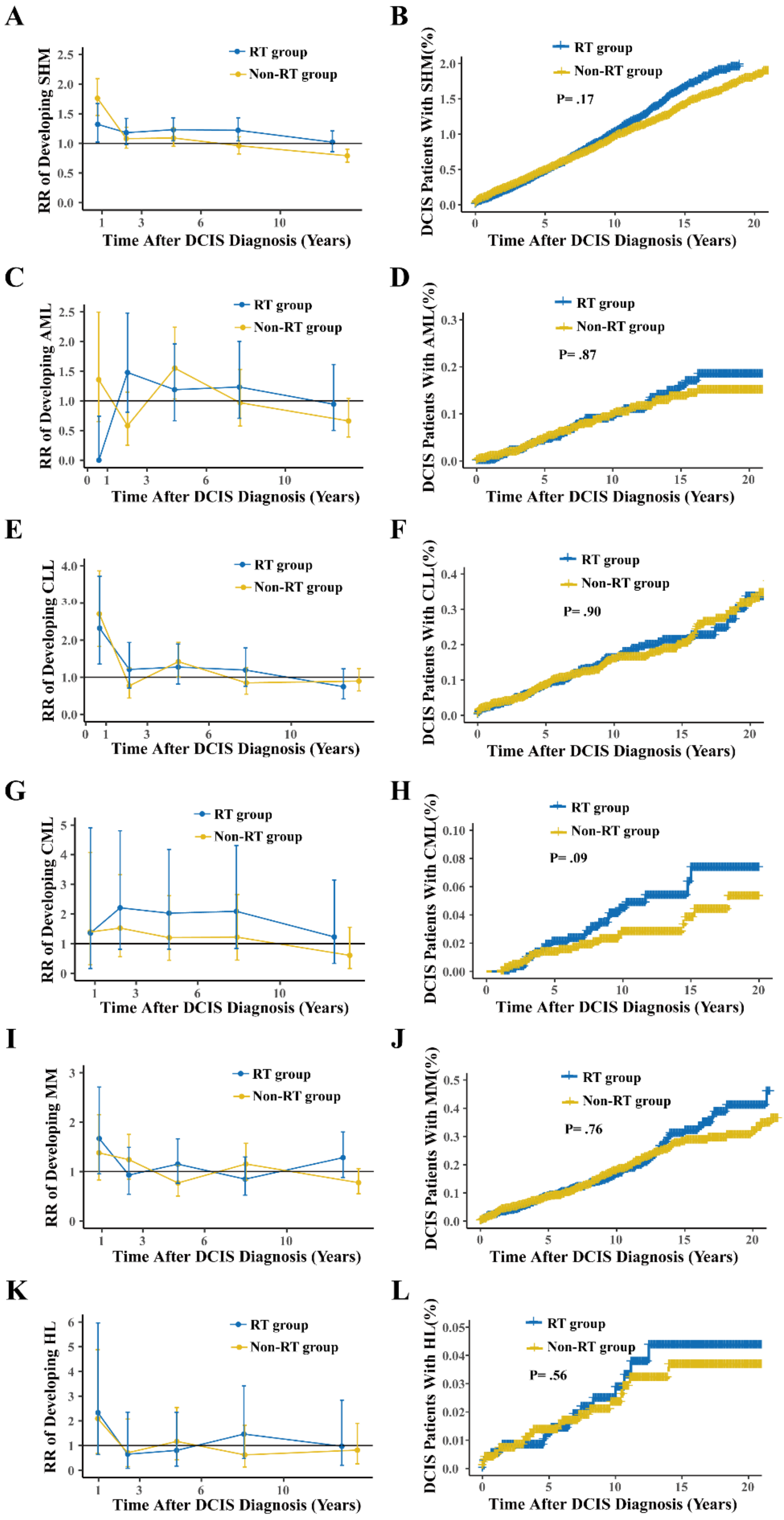

Plotted are mean relative risks (RRs)±CIs of developing (A, B) all SHMs, (C, D) AML, (E, F) CLL, (G, H) CML, (I, J) MM, and (K, L) HL as second cancer, on the basis of DCIS treatment type compared with the background US population, which is represented by the black line at y = 1. The number of expected and observed cases, RRs and 95% CIs for each RR time-course graph are shown in Supplementary Tables 11-18. Patients were censored at death, when they were alive at January 1, 2017, or when they developed a non-SHM second cancer. P values were calculated using the log-rank test.

Supplementary Figure 4: Risk-Time Courses of SHMs After Low/intermediate-risk DCIS Diagnosis.

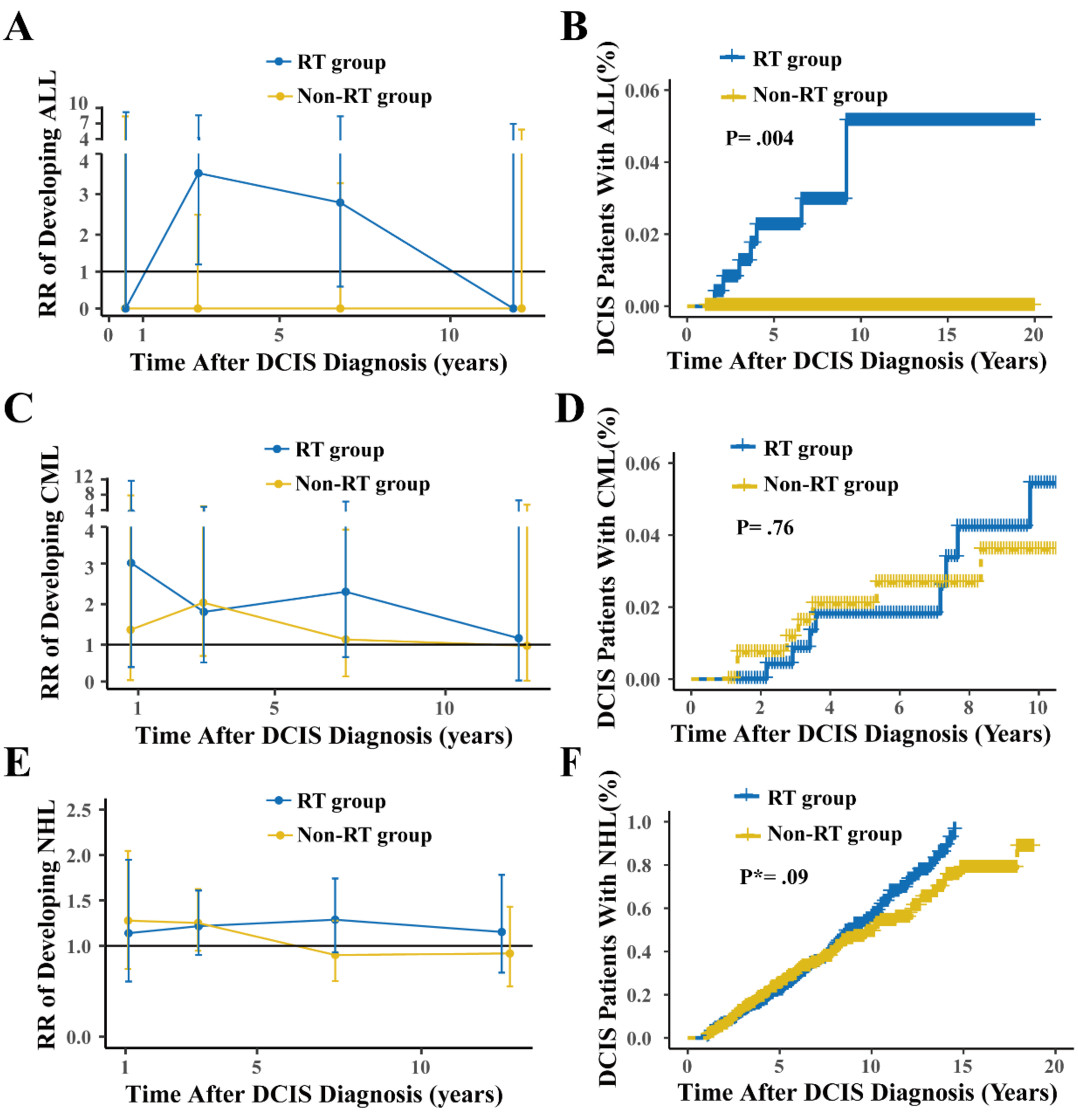

Plotted are mean relative risks (RRs)±CIs of developing (A, B) ALL, (C, D) CML, and (E, F) NHL as second cancer, on the basis of DCIS treatment type compared with the background US population, which is represented by the black line at y = 1. The number of expected and observed cases, RRs and 95% CIs for each RR time-course graph are shown in Supplementary Tables 19-21. Patients were censored at death, when they were alive at January 1, 2017, or when they developed a non-SHM second cancer. P values were calculated using the log-rank test.

*P value was calculated using two-stage procedure test.

Supplementary Figure 5: Risk-Time Courses of SHMs After DCIS Treatment by Age.

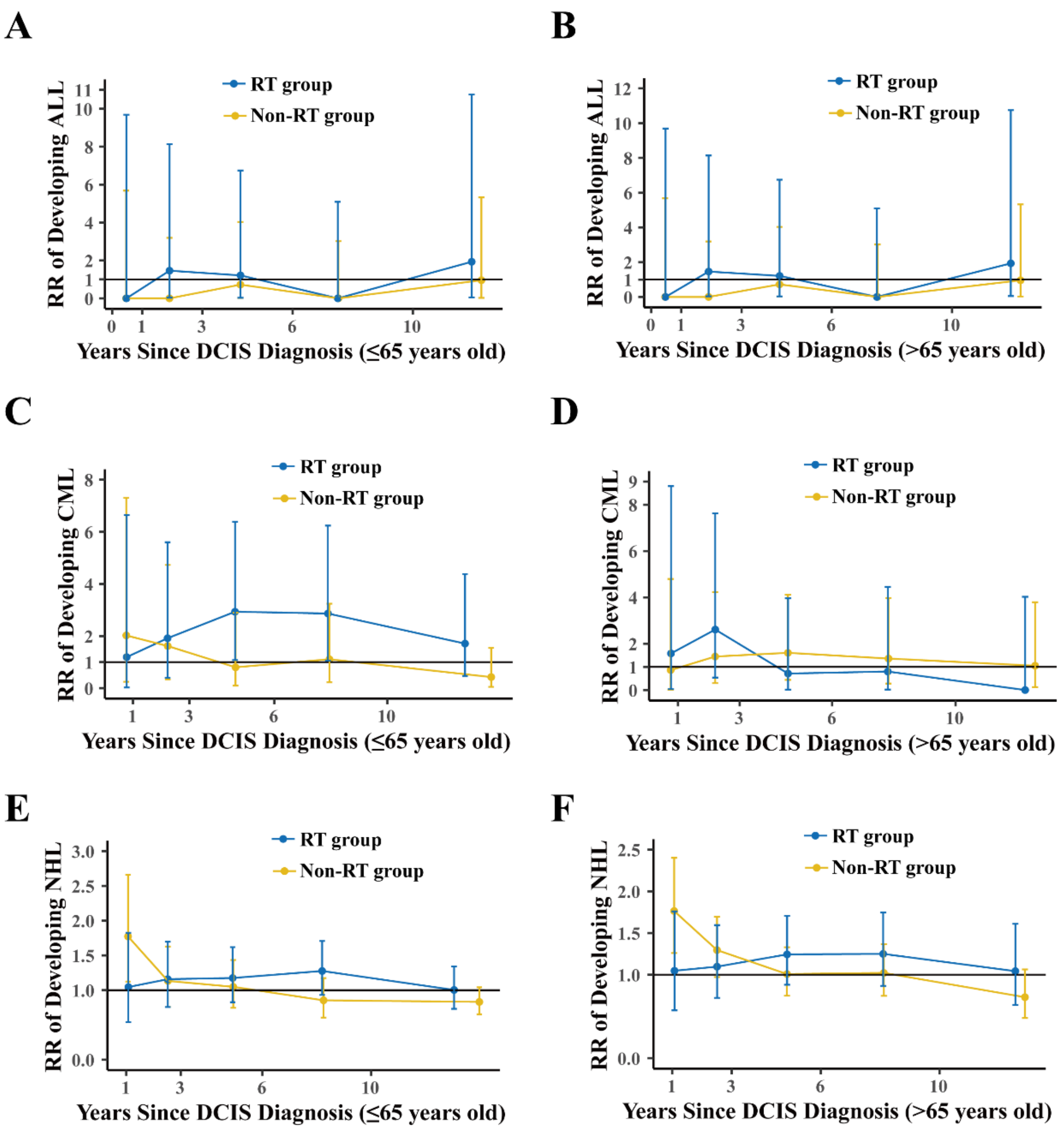

(A, C and E) Data for DCIS patients aged more than 65; (B, D and F) data for DCIS patients aged less than 65. Plotted are mean relative risks (RRs) ± CIs of developing (A, B) ALL, (C, D) CML or (E, F) NHL as second cancer, on the basis of DCIS treatment type compared with the background US population, which is represented by the black line at y = 1……..

Supplementary Figure 6: Time-to-Event Curves of SHM Development After Treatment for DCIS by Age.

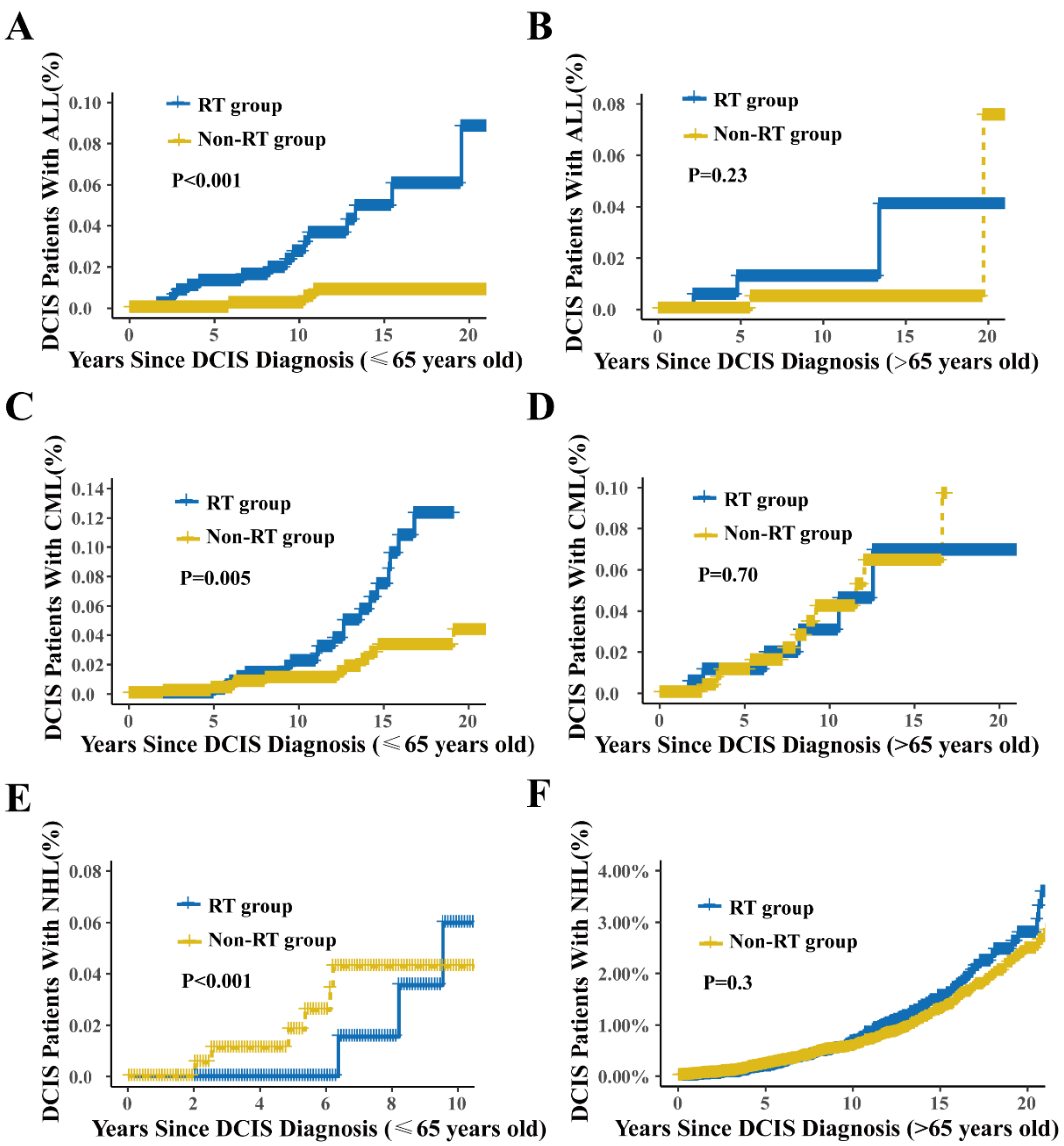

(A, C and E) Data for DCIS aged less than 65; (B, D and F) data for DCIS aged more than 65. Plotted are the percentage of patients with DCIS diagnosed with (A, B) ALL, (C, D) CML and (E, F) NHL as function of the years after DCIS diagnosis. Patients were censored at death, when they were alive at January 1, 2017, or when they developed a non-SHM second cancer. P values were calculated using the Log-rank test.

Supplementary Tables

Supplementary Table 1: ICD Codes of Included ductal carcinoma in situ of the breast and Hematological Malignancies.

Comprehensive Diagnosis	ICD-O-3	Classification
Ductal Carcinoma In Situ of the breast (DCIS) C500-C506, C508-C509	8201	Cribriform carcinoma in situ
	8230	Duct carcinoma in situ, solid type
	8500	Intraductal carcinoma, noninfiltrating, NOS
	8501	Comedocarcinoma, non-infiltrating
	8502	Secretory carcinoma of breast, in situ
	8503	Noninfiltrating intraductal papillary adenocarcinoma
	8504	Noninfiltrating intracystic carcinoma
	8507	Intraductal micropapillary carcinoma
	8523	Intraductal mixed with other types of carcinoma, in situ
Acute lymphoid leukemia (ALL)	9811	B lymphoblastic leukemia/lymphoma, NOS
	9812	Leukemia/lymphoma with t(9;22)(q34;q11.2);BCR-ABL1
	9813	Leukemia/lymphoma with t(v;11q23);MLL rearranged
	9814	Leukemia/lymphoma with t(12;21)(p13;q22);TEL-AML1(ETV6-RUNX1)
	9815	B lymphoblastic leukemia/lymphoma with hyperdiploidy
	9816	Leukemia/lymphoma with hypodiploidy (hypodiploid ALL)
	9817	B lymphblastic leukemia/lymphoma with t(5;14)(q31;q32);IL3-IGH
	9818	Leukemia/lymphoma with t(1;19)(q23;p13.3); E2A PBX1 (TCF3 PBX1)
	9820	Lymphoid leukemia, NOS
	9826	Burkitt cell leukemia
	9827	Adult T-cell leukemia/lymphoma (HTLV-1 pos.)
	9828	Acute lymphoblastic leukemia, L2 type, NOS
	9831	T-cell large granular lymphocytic leukemia (before 2010)
	9832	Prolymphocytic leukemia, NOS
	9833	Prolymphocytic leukemia, B-cell type
	9834	Prolymphocytic leukemia, T-cell type
	9835	Precursor cell lymphoblastic leukemia, NOS
	9836	Precursor B-cell lymphoblastic leukemia
	9837	T lymphoblastic leukemia/lymphoma
Acute myeloid leukemia (AML)	9840	Acute myeloid leukemia, M6 type
	9860	Myeloid leukemia, NOS
	9861	Acute myeloid leukemia
	9865	Acute myeloid leukemia with t(6;9)(p23;q34) DEK-NUP214
	9866	Acute promyelocytic leuk.,t(15;17)(q22;q11-12)
	9867	Acute myelomonocytic leukemia
	9869	Acute myeloid leukemia with inv(3)(q21q26.2) or t(3;3)(q21;q26.2);RPN1EVI1
	9870	Acute basophilic leukemia
	9871	Ac. myelomonocytic leuk. w abn. mar. eosinophils

	9872	Acute myeloid leukemia, minimal differentiation
	9873	Acute myeloid leukemia without maturation
	9874	Acute myeloid leukemia with maturation
	9876	Atypical chronic myeloid leuk., BCR/ABL negative
	9891	Acute monocytic leukemia
	9895	Acute myeloid leuk. with multilineage dysplasia
	9896	Acute myeloid leukemia, t(8;21)(q22;q22)
	9897	Acute myeloid leukemia, 11q23 abnormalities
	9898	Myeloid leukemia associated with Down Syndrome
	9910	Acute megakaryoblastic leukemia
	9911	Acute myeloid leukemia (megakaryoblastic) with t(1;22)(p13;q13);RBM15-MLK1
	9920	Therapy-related acute myeloid leukemia, NOS
	9930	Myeloid sarcoma
	9931	Acute panmyelosis with myelofibrosis
Chronic lymphoid leukemia (CLL)	9823	Chronic lymphocytic leukemia/small lymphocytic lymphoma
Chronic myeloid leukemia (CML)	9863	Chronic myeloid leukemia, NOS
	9875	Chronic myelogenous leukemia, BCR/ABL positive
Hodgkin's lymphoma (HL)	9650	Hodgkin lymphoma, NOS
	9651	Hodgkin lymphoma, lymphocyte-rich
	9652	Hodgkin lymphoma, mixed cellularity, NOS
	9653	Hodgkin lymphoma, lymphocytic deplet., NOS
	9654	Hodgkin lymph., lymphocyt. deplet., diffuse fibrosis
	9655	Hodgkin lymphoma, lymphocyt. deplet., reticular
	9659	Hodgkin lymph., nodular lymphocyte predom.
	9661	Hodgkin granuloma [obs]
	9662	Hodgkin sarcoma [obs]
	9663	Hodgkin lymphoma, nodular sclerosis, NOS
	9664	Hodgkin lymphoma, nod. scler., cellular phase
	9665	Hodgkin lymphoma, nod. scler., grade 1
	9667	Hodgkin lymphoma, nod. scler., grade 2
Non-Hodgkin's lymphoma (NHL)	9590	Malignant lymphoma, NOS
	9591	Malignant lymphoma, non-Hodgkin
	9596	Composite Hodgkin and non-Hodgkin lymphoma
	9670	ML, small B lymphocytic, NOS
	9671	ML, lymphoplasmacytic
	9673	Mantle cell lymphoma
	9675	ML, mixed sm. and lg. cell, diffuse
	9678	Primary effusion lymphoma
	9679	Mediastinal large B-cell lymphoma
	9680	ML, large B-cell, diffuse
	9684	ML, large B-cell, diffuse, immunoblastic, NOS
	9687	Burkitt lymphoma, NOS
	9688	T-cell histiocyte rich large B-cell lymphoma
	9689	Splenic marginal zone B-cell lymphoma
	9690	Follicular lymphoma, NOS

	9691	Follicular lymphoma, grade 2
	9695	Follicular lymphoma, grade 1
	9698	Follicular lymphoma, grade 3
	9699	Marginal zone B-cell lymphoma, NOS
	9701	Sezary syndrome
	9702	Mature T-cell lymphoma, NOS
	9705	Angioimmunoblastic T-cell lymphoma
	9712	Intravascular large B-cell lymphoma
	9714	Anaplastic large cell lymphoma, T-cell and Null cell type
	9719	NK/T-cell lymphoma, nasal and nasal-type
	9724	SystemicEBV pos. T-cell lymphoproliferative disease of childhood
	9727	Precursor cell lymphoblastic lymphoma, NOS
	9728	Precursor B-cell lymphoblastic lymphoma
	9729	Precursor T-cell lymphoblastic lymphoma
	9735	Plasmablastic lymphoma
	9737	ALK positive large B-cell lymphoma
	9738	Lrg B-cell lymphoma in HHV8-assoc. multicentric Castleman DZ
	9971	Polymorphic PTLD
Multiple myeloma (MM)	9731	Plasmacytoma, NOS
	9732	Multiple myeloma
	9733	Plasma cell leukemia
	9734	Plasmacytoma, extramedullary
	9760	Immunoproliferative disease, NOS
	9761	Waldenstrom macroglobulinemia
	9762	Heavy chain disease, NOS

ICD9/10 and ICD-O-3 morphological codes that were used to select DCIS and secondary hematological malignancy cases.

Supplementary Table 2: All Hematologic and Non-Hematologic Second Cancers Identified After DCIS Diagnoses.

	Non-RT (n = 106,436)	RT (n = 77,927)
**All SHM cases combined**	857	629
Months to development of any SHM, median (IQR)	64 (24, 118)	66 (32, 112)
**ALL cases**	7	19
Months to development of ALL, median (IQR)	85 (36, 152)	85 (39, 140)
**AML cases**	82	58
Months to development of AML, median (IQR)	63 (39, 111)	71 (40, 96)
**CLL cases**	146	96
Months to development of CLL, median (IQR)	62 (23, 122)	54 (24, 97)
**CML cases**	25	26
Months to development of CML, median (IQR)	42 (23, 100)	55 (35, 105)
**MM cases**	156	115
Months to development of MM, median (IQR)	74 (21, 118)	69 (34, 128)
**HL cases**	22	17
Months to development of HL, median (IQR)	62 (16, 100)	45 (15, 111)
**NHL cases**	419	298
Months to development of NHL, median (IQR)	65 (24, 119)	70 (33, 113)
**Other leukemias**	151	102
Months to development of other leukemia, median (IQR)	72 (28, 144)	86 (32, 132)
**Breast cancer**	11,836	6,859
Months to development of breast cancer, median (IQR)	40 (4, 93)	67 (27, 116)
**Solid tumors other than breast cancer**	6,622	4,351
Months to development of solid tumor, median (IQR)	66 (27, 124)	66 (29, 111)

Follow-up times are in years. Abbreviations: DCIS, ductal carcinoma in situ of the breast; RT, radiotherapy; ALL, acute lymphocytic leukemia; AML, acute myeloid leukemia; CLL, chronic lymphocytic leukemia; CML, chronic myeloid leukemia; HL, Hodgkin lymphoma; IQR, interquartile range; MM, multiple myeloma; NHL, non-Hodgkin lymphoma.

Supplementary Table 3: Baseline characteristics of DCIS patients who later developed ALL.

Patient characteristic		DCIS cases later developed ALL			DCIS cases did not develop ALL			
		Non-RT (n = 7)	RT (n = 19)	P value*	Non-RT (n = 106,429)	P value**	RT (n = 77,908)	P value***
Age at DCIS diagnosis	0-29 years	0 (0.0)	0 (0.0)	0.70 M	380 (0.4)	0.39 M	81(0.1)	0.66 M
	30-39 years	1 (14.3)	1 (5.3)		4,392 (4.1)		1,923 (2.5)	
	40-49 years	2 (28.6)	4 (21.1)		23,540 (22.1)		17,071 (21.9)	
	50-59 years	0 (0.0)	4 (21.1)		27,659 (26.0)		23,912 (30.7)	
	60-69 years	3 (42.9)	8 (42.1)		24,405 (22.9)		21,315 (27.4)	
	≥ 70 years	1 (14.3)	2 (10.5)		26,053 (24.5)		13,606 (17.5)	
Year of DCIS diagnosis	1975-1979	0 (0.0)	0 (0.0)	0.64 F	1,053 (1.0)	0.15 F	36 (0.0)	<0.001F
	1980-1989	1 (14.3)	1 (5.3)		5,314 (5.0)		1016 (1.3)	
	1990-1999	3 (42.9)	8 (42.1)		17,151 (16.1)		8,674 (11.1)	
	2000-2009	3 (42.9)	7 (36.8)		46,722 (43.9)		37,386 (48.0)	
	2010-2016	0 (0.0)	3 (15.8)		36,189 (34.0)		30,796 (39.5)	
Race	White	7 (100.0)	13 (68.4)	0.24 F	82,947 (77.9)	0.37 F	60,706 (77.9)	0.61 F
	Black	0 (0.0)	3 (15.8)		11,021 (10.4)		8,411 (10.8)	
	Other	0 (0.0)	3 (15.8)		12,461 (11.7)		8,791 (11.3)	
Tumor size	<1cm	1 (14.3)	7 (36.8)	0.04 F	32,330 (30.4)	0.17 F	27,618 (35.4)	0.91 F
	1-2 cm	0 (0.0)	5 (26.3)		16,426 (15.4)		16,825 (21.6)	
	2-5 cm	0 (0.0)	3 (15.8)		15,018 (14.1)		10,280 (13.2)	
	>5cm	1 (14.3)	0 (0.0)		5,935 (5.6)		1,748 (2.2)	
	Unknown	5 (71.4)	4 (21.1)		36,720 (34.5)		21,437 (27.5)	
Grade	I	0 (0.0)	2 (10.5)	0.11 F	12,238 (11.5)	0.08 F	8,092 (10.4)	0.71F
	II	1 (14.3)	4 (21.1)		32,122 (30.2)		26,034 (33.4)	
	III	1 (14.3)	9 (47.4)		32,689 (30.7)		29,901 (38.4)	
	Unknown	5 (71.4)	4 (21.1)		29,380 (27.6)		13,881 (17.8)	
ER	Negative	1 (14.3)	3 (15.8)	0.64 F	8,992 (8.4)	0.25 F	7,673 (9.8)	0.07F
	Positive	1 (14.3)	6 (31.6)		48,664 (45.7)		45,237 (58.1)	
	Unknown	5 (71.4)	10 (52.6)		48,773 (45.8)		24,998 (32.1)	
PR	Negative	1 (14.3)	3 (15.8)	0.64 F	13,529 (12.7)	0.43 F	11,918 (15.3)	0.30 F
	Positive	1 (14.3)	6 (31.6)		40,053 (37.6)		37,468 (48.1)	
	Unknown	5 (71.4)	10 (52.6)		52,847 (49.7)		28,522 (36.6)	
Surgery	No	0 (0.0)	0 (0.0)	0.01 F	4,355 (4.1)	0.30 F	389 (0.5)	0.93F
	BCS	4 (57.1)	19 (100.0)		46,666 (43.8)		76,035 (97.6)	
	Mastectomy	1 (14.3)	0 (0.0)		43,528 (40.9)		1,199 (1.5)	

	**Unknown**	2 (28.6)	0 (0.0)		11880 (11.2)		285 (0.4)	
**Median PYs of follow-up (IQR)**		5.9 (4.1,13.3)	4 (2.1, 8.5)	0.36^{M}	7.1 (3, 12.7)	0.91^{M}	7.1 (3.3, 11.7)	0.87^{M}

Percentages shown are calculated within rows. *Concerns a comparison of DCIS patients treated with RT that later developed ALL versus DCIS patients without RT that later developed ALL. **Concerns a comparison of DCIS patients without RT that later developed ALL versus counterparts that did not develop ALL. ***Concerns a comparison of DCIS patients treated with RT that later developed ALL versus counterparts that did not develop ALL. M Mann-Whitney U test. F Fisher’s exact test.

Abbreviations: IQR, interquartile range; ALL, acute lymphocytic leukemia; RT, radiotherapy; PY, person-year; DCIS, ductal carcinoma in situ of the breast.

Supplementary Table 4: Baseline characteristics of DCIS patients who later developed CML.

Patient characteristic		DCIS cases later developed CML			DCIS cases did not develop CML			
		Non-RT (n = 25)	RT (n = 26)	P value*	Non-RT (n = 106,411)	P value**	RT (n = 77,901)	P value***
Age at DCIS diagnosis	0-29 years	0 (0.0)	0 (0.0)	0.02 ^{M}	380 (0.4)	0.21 ^{M}	81 (0.1)	0.86 ^{M}
	30-39 years	0 (0.0)	0 (0.0)		4,393(4.1)		1,924 (2.5)	
	40-49 years	7 (28.0)	5 (19.2)		23,535(22.1)		17,070(21.9)	
	50-59 years	2 (8.0)	10(38.5)		27,657(26.0)		23,906 (30.7)	
	60-69 years	6 (24.0)	8 (30.8)		24,402(22.9)		21,315 (27.4)	
	≥ 70 years	10 (40.0)	3 (11.5)		26,044(24.5)		13,605 (17.5)	
Year of DCIS diagnosis	1975-1979	2 (8.0)	0 (0.0)	0.21 ^{F}	1,051(1.0)	0.004 ^{F}	36 (0.0)	0.15 ^{F}
	1980-1989	2 (8.0)	0 (0.0)		5,313(5.0)		1,017 (1.3)	
	1990-1999	4 (16.0)	2 (7.7)		17,150 (16.1)		8,680 (11.1)	
	2000-2009	13 (52.0)	19 (73.1)		46,712 (43.9)		37,374 (48.0)	
	2010-2016	4 (16.0)	5 (19.2)		36,185(34.0)		30,794 (39.5)	
Race	White	22 (88.0)	23 (88.5)	0.99 ^{F}	82,932 (77.9)	0.42 ^{F}	60,696 (77.9)	0.39 ^{F}
	Black	2 (8.0)	2 (7.7)		11,019 (10.4)		8,412 (10.8)	
	Other	1 (4.0)	1 (3.8)		12460 (11.7)		8,793 (11.3)	
Tumor size	<1cm	7 (28.0)	10 (38.5)	0.57 ^{F}	32,324(30.4)	0.56 ^{F}	27,615 (35.4)	0.44 ^{F}
	1-2 cm	3 (12.0)	5 (19.2)		16,423(15.4)		16,825 (21.6)	
	2-5 cm	5 (20.0)	6 (23.1)		15,013(14.1)		10,277 (13.2)	
	>5cm	3 (12.0)	1 (3.8)		5,933 (5.6)		1,747 (2.2)	
	Unknown	7 (28.0)	4 (15.4)		36,718 (34.5)		21,437 (27.5)	
Grade	I	2 (8.0)	3 (11.5)	0.61 ^{F}	12,236 (11.5)	0.73 ^{F}	8,091 (10.4)	0.88 ^{F}
	II	8 (32.0)	10 (38.5)		32,115 (30.2)		26,028 (33.4)	
	III	6 (24.0)	8 (30.8)		32,684 (30.7)		29,902 (38.4)	
	Unknown	9 (36.0)	5 (19.2)		29,376 (27.6)		13,880 (17.8)	
ER	Negative	2 (8.0)	5 (19.2)	0.49 ^{F}	8,991(8.4)	0.58 ^{F}	7,671 (9.8)	0.04 ^{F}
	Positive	9 (36.0)	9 (34.6)		48,656 (45.7)		45,234 (58.1)	
	Unknown	14 (56.0)	12 (46.2)		48,764(45.8)		24,996 (32.1)	
PR	Negative	2 (8.0)	6 (23.1)	0.14 ^{F}	13,528 (12.7)	0.08 ^{F}	11,915 (15.3)	0.19 ^{F}
	Positive	5 (20.0)	8 (30.8)		40,049 (37.6)		37,466 (48.1)	
	Unknown	18 (72.0)	12 (46.2)		52,834 (49.7)		28,520 (36.6)	
Surgery	No	0 (0.0)	0 (0.0)	<0.001 ^{F}	4,355(4.1)	0.05 ^{F}	389(0.5)	0.89 ^{F}
	BCS	9 (36.0)	26(100.0)		46,661 (43.8)		76,028(97.6)	
	Mastectomy	9 (36.0)	0 (0.0)		43,520 (40.9)		1,199(1.5)	

	**Unknown**	7 (28.0)	0 (0.0)		11,875 (11.2)		285(0.4)	
**Median PYs of follow-up (IQR)**		3.5 (1.9, 8.3)	4.6 (2.9, 8.8)	0.45^{M}	7.1 (3.0, 12.7)	0.05^{M}	7.1 (3.3, 11.7)	0.05^{M}

Percentages shown are calculated within rows. *Concerns a comparison of DCIS patients treated with RT that later developed CML *versus* DCIS patients without RT that later developed CML. **Concerns a comparison of DCIS patients without RT that later developed CML *versus* counterparts that did not develop CML. ***Concerns a comparison of DCIS patients treated with RT that later developed CML *versus* counterparts that did not develop CML. ^{M} Mann-Whitney *U* test. ^{F} Fisher's exact test.

Abbreviations: IQR, interquartile range; CML, chronic myeloid leukemia; RT, radiotherapy; PY, person-year; DCIS, ductal carcinoma in situ of the breast.

Supplementary Table 5: Baseline characteristics of DCIS patients who later developed NHL.

Patient characteristic		DCIS cases later developed NHL			DCIS cases did not develop NHL			
		Non-RT (n = 417)	RT (n = 298)	P value*	Non-RT (n = 106,017)	P value**	RT (n = 77,629)	P value***
Age at DCIS diagnosis	0-29 years	1 (0.2)	0 (0.0)		379 (0.4)		81(0.1)	
	30-39 years	8 (1.9)	1 (0.3)		4,385 (4.1)		1,923(2.5)	
	40-49 years	53 (12.6)	31 (10.4)		23,489(22.2)		17,044(22.0)	
	50-59 years	79 (18.9)	61 (20.5)	0.02[M]	27,580 (26.0)	<0.001[M]	23,855(30.7)	<0.001[M]
	60-69 years	115 (27.4)	112 (37.6)		24,293(22.9)		21,211(27.3)	
	≥ 70 years	163 (38.9)	93 (31.2)		25,891(24.4)		13,515(17.4)	
Year of DCIS diagnosis	1975-1979	8 (1.9)	0 (0.0)		1,045(1.0)		36(0.0)	
	1980-1989	47 (11.2)	6 (2.0)		5,268(5.0)		1,011(1.3)	
	1990-1999	108 (25.8)	67 (22.5)	<0.001[F]	17,046(16.1)	<0.001[F]	8,615(11.1)	<0.001[F]
	2000-2009	214 (51.1)	185 (62.1)		46,511(43.9)		37,208(47.9)	
	2010-2016	42 (10.0)	40 (13.4)		36,147(34.1)		30,759(39.6)	
Race	White	355 (84.7)	251 (84.2)		82,599(77.9)		60,468(77.9)	
	Black	27 (6.4)	25 (8.4)	0.51[F]	10,994(10.4)	0.003[F]	8,389(10.8)	0.03[F]
	Other	37 (8.8)	22 (7.4)		12,424(11.7)		8,772(11.3)	
Tumor size	<1cm	145 (34.6)	119 (39.9)		32,186(30.4)		27,506 (35.4)	
	1-2 cm	63 (15.0)	68 (22.8)		16,363(15.4)		16,762 (21.6)	
	2-5 cm	45 (10.7)	36 (12.1)	0.001[F]	14,973(14.1)	0.08[F]	10,247(13.2)	0.33[F]
	>5cm	16 (3.8)	7 (2.3)		5,920(5.6)		1,741 (2.2)	
	Unknown	150 (35.8)	68 (22.8)		36,575(34.5)		21,373(27.5)	
Grade	I	45 (10.7)	24 (8.1)		12,193(11.5)		8,070 (10.4)	
	II	94 (22.4)	86 (28.9)	0.02[F]	32,029(30.2)	<0.001[F]	25,952(33.4)	<0.001[F]
	III	118 (28.2)	101 (33.9)		32,572(30.7)		29,809(38.4)	
	Unknown	162 (38.7)	87 (29.2)		29,223(27.6)		13,798(17.8)	
ER	Negative	27 (6.4)	28 (9.4)		8,966(8.5)		7,648(9.9)	
	Positive	117 (27.9)	132 (44.3)	<0.001[F]	48,548(45.8)	<0.001[F]	45,111(58.1)	<0.001[F]
	Unknown	275 (65.6)	138 (46.3)		48,503(45.8)		24,870 (32.0)	
PR	Negative	46 (11.0)	43 (14.4)		13,484(12.7)		11,878 (15.3)	
	Positive	85 (20.3)	108 (36.2)	<0.001[F]	39,969(37.7)	<0.001[F]	37,366 (48.1)	<0.001[F]
	Unknown	288 (68.7)	147 (49.3)		52,564 (49.6)		28,385(36.6)	
Surgery	No	15 (3.6)	2 (0.7)		4,340 (4.1)		387 (0.5)	
	BCS	189 (45.1)	290 (97.3)	<0.001[F]	46,481(43.8)	<0.001[F]	75,764 (97.6)	0.80[F]
	Mastectomy	133 (31.7)	4 (1.3)		43,396(40.9)		1,195 (1.5)	

	**Unknown**	82 (19.6)	2 (0.7)		11,800(11.1)		283 (0.4)	
**Median PYs of follow-up (IQR)**		5.4 (2.0, 9.9)	5.8 (2.8, 9.4)	0.24 [M]	5.4 (2.0, 9.9)	<0.001 [M]	7.1 (3, 12.7)	<0.001 [M]

Percentages shown are calculated within rows. *Concerns a comparison of DCIS patients treated with RT that later developed NHL *versus* DCIS patients without RT that later developed NHL. **Concerns a comparison of DCIS patients without RT that later developed NHL *versus* counterparts that did not develop NHL. ***Concerns a comparison of DCIS patients treated with RT that later developed NHL *versus* counterparts that did not develop NHL. [M] Mann-Whitney U test. [F] Fisher's exact test.

Abbreviations: IQR, interquartile range; NHL, non-Hodgkin lymphoma; RT, radiotherapy; PY, person-year; DCIS, ductal carcinoma in situ of the breast

Supplementary Table 6. Univariable Competing Risk Regression Analysis of Risk of Developing Hematologic Malignancies in subjects with DCIS.

**Covariables**	**ALL**		**AML**		**CLL**		**CML**		**MM**		**HL**		**NHL**		**SHMs Combined**	
	**HR (95% CI)**	**P**	**HR (95% CI)**	**P**	**HR (95% CI)**	**P**	**HR (95% CI)**	**P**	**HR (95% CI)**	**P**	**HR (95% CI)**	**P**	**HR (95% CI)**	**P**	**HR (95% CI)**	**P**
**Age, per year**	0.99 [0.96, 1.02]	0.67	**1.04 [1.03, 1.05]**	**<0.001**	**1.04 [1.03, 1.05]**	**<0.001**	1.01 [0.99, 1.03]	0.21	**1.03 [1.02, 1.04]**	**<0.001**	1.01 [0.99,1.03]	0.47	**1.03 [1.03, 1.04]**	**<0.001**	**1.03 [1.03, 1.04]**	**<0.001**
**Year of diagnosis, per year**	**0.95 [0.91, 1.00]**	**0.03**	0.98 [0.96, 1.01]	0.18	**0.97 [0.95, 0.98]**	**<0.001**	1.02 [0.98, 1.07]	0.3	0.99 [0.98, 1.01]	0.27	0.97 [0.94, 1.01]	0.14	**0.99[0.98, 1.00]**	**0.05**	**0.99 [0.98, 0.99]**	**<0.001**
**Race:** Black vs White	1.28 [0.38, 4.36]	0.69	1.09 [0.62,1.91]	0.76	0.78 [0.47, 1.30]	0.34	0.80 [0.29, 2.21]	0.66	**1.93 [1.36, 2.75]**	**<0.001**	/	/	**0.70 [0.52, 0.95]**	**0.02**	0.96 [0.79, 1.15]	0.64
**Race:** Others vs White	1.23 [0.36, 4.17]	0.74	0.52[0.24, 1.13]	0.10	**0.14 [0.04, 0.44]**	**<0.001**	0.38 [0.09, 1.58]	0.18	1.42 [0.95, 2.1]	0.08	/	/	0.83 [0.62, 1.09]	0.18	**0.74 [0.61, 0.92]**	**0.005**
**Tumor size：** 10-19 vs 1-9mm	1.21 [0.40, 3.67]	0.74	1.01 [0.62, 1.63]	0.98	0.91 [0.60, 1.37]	0.65	0.85[0.35, 2.05]	0.71	1.17 [0.81, 1.69]	0.41	/	/	0.93 [0.74, 1.16]	0.52	0.98 [0.84, 1.15]	0.83
**Tumor size：** 20-49 vs 1-9 mm	0.67 [0.14, 3.15]	0.61	0.67 [0.36, 1.27]	0.22	0.77 [0.47, 1.27]	0.30	1.68 [0.76, 3.71]	0.20	0.95 [0.61, 1.48]	0.81	/	/	**0.79 [0.60, 1.03]**	**0.09**	**0.83 [0.68, 1.00]**	**0.05**
**Tumor size：** 50+ vs 1-9 mm	1.16 [0.14, 9.33]	0.89	0.39 [0.09, 1.59]	0.19	0.80 [0.35, 1.84]	0.60	2.33 [0.78, 6.98]	0.13	1.02 [0.79, 1.49]	0.97	/	/	0.85 [0.54, 1.33]	0.47	0.86 [0.63, 1.18]	0.36
**Grade：** II vs I	0.90 [0.17, 4.68]	0.90	1.08 [0.56, 2.07]	0.82	0.94 [1.07, 0.53]	0.81	1.53 [0.51, 4.55]	0.44	0.76 [0.46, 1.26]	0.28	1.80 [0.21,15.4]	0.59	0.90 [0.67, 1.21]	0.48	0.92 [0.75, 1.14]	0.46
**Grade：** III vs I	1.47 [0.32, 6.83]	0.62	0.86 [0.44, 1.68]	0.66	1.05 [0.95, 0.61]	0.85	1.06 [0.34, 3.24]	0.92	1.17 [0.73, 1.88]	0.52	2.9 [0.37, 22.9]	0.31	1.01 [0.76, 1.35]	0.93	1.06 [0.86, 1.30]	0.60
**ER**: positive vs negative	**0.30 [0.09, 1.08]**	**0.07**	1.29 [0.61, 2.72]	0.51	**0.58 [0.33, 1.01]**	**0.06**	0.47 [0.19, 1.12]	0.10	1.17 [0.66, 2.07]	0.58	/	/	0.82 [0.60, 1.11]	0.19	0.84[0.68, 1.04]	0.11
**PR**: positive vs negative	0.56 [0.16, 2.02]	0.38	0.92 [0.52, 1.65]	0.79	0.87 [0.51, 1.49]	0.62	0.50 [0.20, 1.24]	0.14	1.06 [0.66, 1.71]	0.79	/	/	**0.71 [0.54, 0.93]**	**0.01**	**0.81 [0.67, 0.98]**	**0.03**
**Surgery:** mastectomy vs BCS	**0.14 [0.02, 1.02]**	**0.06**	0.83 [0.53, 1.31]	0.43	**0.68 [0.46, 1.02]**	**0.06**	0.72 [0.33, 1.56]	0.40	0.89 [0.64, 1.24]	0.48	0.59 [0.20, 1.71]	0.33	**0.82 [0.67, 1.01]**	**0.06**	**0.79 [0.68, 0.91]**	**0.01**
**Treatment**: RT vs Non-RT	**3.86 [1.64, 9.10]**	**0.002**	1.27 [0.89, 1.80]	0.19	1.12 [0.84, 1.50]	0.44	**1.69 [0.97, 3.00]**	**0.06**	1.13 [0.87, 1.47]	0.37	1.21 [0.58, 2.51]	0.62	**1.19 [1.01, 1.39]**	**0.03**	**1.22 [1.09, 1.36]**	**<0.001**

NOTE. Shown are HRs and 95% CIs for developing a nonsynchronous (⩾1 year after DCIS diagnosis) SHM in patients with DCIS, calculated using Fine-Gray competing risk regression analyses.

Bold type indicates the numbers that were significant in univariate analysis.

Abbreviations: ALL, acute lymphocytic leukemia; AML, acute myeloid leukemia; CLL, chronic lymphocytic leukemia; CML, chronic myeloid leukemia; HL, Hodgkin lymphoma; HR, hazard ratio; MM, multiple myeloma; NHL, non-Hodgkin lymphoma; SHM, second hematologic malignancy; DCIS, ductal carcinoma in situ of the breast.

Supplementary Table 7: Relative Risk-Time Course Specifics for Each Time Interval: Any SHM After DCIS dignosis.

	Non-RT					RT				
**Interval**	**O**	**E**	**RR**	**95% CI**	**P**	**O**	**E**	**RR**	**95% CI**	**P**
**0 - 1**	130	73.70	1.76	1.47-2.09	<0.001	67	50.85	1.32	1.02-1.67	0.02
**1 - 3**	149	137.39	1.08	0.92-1.27	0.32	114	96.40	1.18	0.98-1.42	0.07
**3 - 6**	196	179.23	1.09	0.95-1.26	0.21	156	127.21	1.23	1.04-1.43	0.01
**6 - 10**	175	182.75	0.96	0.82-1.11	0.57	157	128.41	1.22	1.04-1.43	0.01
**>10**	207	262.81	0.79	0.68-0.90	<0.001	135	132.14	1.02	0.86-1.21	0.80

See below for legend.

Supplementary Table 8: Relative Risk-Time Course Specifics for Each Time Interval: ALL After DCIS dignosis.

	Non-RT					RT				
**Interval**	**O**	**E**	**RR**	**95% CI**	**P**	**O**	**E**	**RR**	**95% CI**	**P**
**0 - 1**	0	1.29	0	0-2.87	0.26	2	0.94	2.13	0.26-7.70	0.27
**1 - 3**	2	2.36	0.85	0.10-3.07	0.81	5	1.73	2.89	0.94-6.74	0.05
**3 - 6**	2	3.01	0.67	0.08-2.40	0.56	4	2.21	1.81	0.49-4.64	0.48
**6 - 10**	1	2.99	0.34	0.008-1.87	0.25	6	2.14	2.80	1.03-6.10	0.03
**>10**	2	4.03	0.50	0.06-1.79	0.31	2	2.07	0.97	0.12-3.50	0.99

See below for legend.

Supplementary Table 9: Relative Risk-Time Course Specifics for Each Time Interval: AML After DCIS dignosis.

	Non-RT					RT				
**Interval**	**O**	**E**	**RR**	**95% CI**	**P**	**O**	**E**	**RR**	**95% CI**	**P**
**0 - 1**	10	7.37	1.36	0.65-2.50	0.63	0	4.98	0	0-0.74	0.03
**1 - 3**	8	13.77	0.58	0.25-1.15	0.30	14	9.47	1.48	0.81-2.48	0.14
**3 - 6**	28	18.05	1.55	1.03-2.24	0.02	15	12.62	1.19	0.67-1.96	0.50
**6 - 10**	18	18.59	0.97	0.57-1.53	0.89	16	12.97	1.23	0.71-2.00	0.40
**>10**	18	27.25	0.66	0.39-1.04	0.08	13	13.78	0.94	0.50-1.61	0.83

See below for legend.

Supplementary Table 10: Relative Risk-Time Course Specifics for Each Time Interval: CLL After DCIS dignosis.

	Non-RT					RT				
**Interval**	**O**	**E**	**RR**	**95% CI**	**P**	**O**	**E**	**RR**	**95% CI**	**P**
**0 - 1**	30	11.07	2.71	1.83-3.87	<0.001	17	7.32	2.32	1.35-3.72	<0.001
**1 - 3**	16	20.84	0.77	0.44-1.25	0.29	17	14.05	1.21	0.71-1.94	0.43
**3 - 6**	39	27.48	1.42	1.01-1.94	0.03	24	18.84	1.27	0.82-1.90	0.23
**6 - 10**	24	28.29	0.85	0.54-1.26	0.42	23	19.28	1.19	0.76-1.79	0.40
**>10**	37	41.34	0.90	0.63-1.23	0.50	15	20.17	0.74	0.42-1.23	0.25

See below for legend.

Supplementary Table 11: Relative Risk-Time Course Specifics for Each Time Interval: CML After DCIS dignosis.

	Non-RT					RT				
**Interval**	**O**	**E**	**RR**	**95% CI**	**P**	**O**	**E**	**RR**	**95% CI**	**P**
**0 - 1**	3	2.15	1.40	0.29-4.08	0.56	2	1.47	1.36	0.17-4.91	0.66
**1 - 3**	6	3.93	1.53	0.56-3.32	0.30	6	2.71	2.21	0.81-4.81	0.05
**3 - 6**	6	4.98	1.20	0.44-2.62	0.65	7	3.45	2.03	0.82-4.18	0.06
**6 - 10**	6	4.91	1.22	0.45-2.66	0.62	7	3.35	2.09	0.84-4.31	0.05
**>10**	4	6.59	0.61	0.17-1.56	0.31	4	3.25	1.23	0.34-3.15	0.68

See below for legend.

Supplementary Table 12: Relative Risk-Time Course Specifics for Each Time Interval: MM After DCIS dignosis.

	Non-RT					RT				
**Interval**	**O**	**E**	**RR**	**95% CI**	**P**	**O**	**E**	**RR**	**95% CI**	**P**
**0 - 1**	19	13.78	1.38	0.83-2.15	0.16	16	9.58	1.67	0.95-2.71	0.05
**1 - 3**	32	25.77	1.24	0.85-1.75	0.22	17	18.28	0.93	0.54-1.49	0.76
**3 - 6**	26	33.77	0.77	0.50-1.13	0.18	28	24.32	1.15	0.77-1.66	0.46
**6 - 10**	40	34.65	1.15	0.83-1.57	0.36	21	24.78	0.85	0.53-1.30	0.45
**>10**	39	50.25	0.78	0.55-1.06	0.11	33	25.75	1.28	0.88-1.80	0.15

See below for legend.

Supplementary Table 13: Relative Risk-Time Course Specifics for Each Time Interval: HL After DCIS dignosis.

	Non-RT					RT				
**Interval**	**O**	**E**	**RR**	**95% CI**	**P**	**O**	**E**	**RR**	**95% CI**	**P**
**0 - 1**	5	2.39	2.09	0.68-4.88	0.09	4	1.72	2.33	0.63-5.96	0.08
**1 - 3**	3	4.24	0.71	0.15-2.07	0.55	2	3.08	0.65	0.08-2.35	0.54
**3 - 6**	6	5.14	1.17	0.43-2.54	0.70	3	3.74	0.80	0.17-2.35	0.70
**6 - 10**	3	4.83	0.62	0.13-1.82	0.41	5	3.42	1.46	0.48-3.42	0.39
**>10**	5	6.13	0.82	0.27-1.90	0.65	3	3.09	0.97	0.2-2.84	0.96

See below for legend.

Supplementary Table 14: Relative Risk-Time Course Specifics for Each Time Interval: NHL After DCIS dignosis.

	Non-RT					RT				
**Interval**	**O**	**E**	**RR**	**95% CI**	**P**	**O**	**E**	**RR**	**95% CI**	**P**
**0 - 1**	63	35.65	1.77	1.36-2.26	<0.001	26	24.84	1.05	0.68-1.53	0.82
**1 - 3**	82	66.50	1.23	0.98-1.53	0.06	53	47.07	1.13	0.84-1.47	0.39
**3 - 5**	58	59.75	0.97	0.74-1.26	0.82	45	42.68	1.05	0.77-1.41	0.72
**5 - 7**	54	52.15	1.04	0.78-1.35	0.80	50	37.30	1.34	1.00-1.77	0.04
**7-10**	60	63.39	0.95	0.72-1.22	0.67	59	44.53	1.33	1.01-1.71	0.03
**>10**	102	127.22	0.80	0.65-0.97	0.03	65	64.03	1.02	0.78-1.29	0.90

See below for legend.

Supplementary Table 15: Relative Risk-Time Course Specifics for Each Time Interval: ALL After Low/Intermediate-Risk DCIS dignosis.

	Non-RT					RT				
**Interval**	**O**	**E**	**RR**	**95% CI**	**P**	**O**	**E**	**RR**	**95% CI**	**P**
**0 - 1**	0	0.44	0	0-8.36	0.51	0	0.40	0	0-9.20	0.53
**1 - 5**	0	1.45	0	0-2.54	0.23	5	1.36	3.67	1.19-8.56	0.002
**5- 10**	0	1.09	0	0-3.39	0.30	3	1.05	2.87	0.59-8.39	0.06
**>10**	0	0.64	0	0-5.81	0.42	0	0.53	0	0-6.93	0.47

See below for legend.

Supplementary Table 16: Relative Risk-Time Course Specifics for Each Time Interval: CML After Low/Intermediate-Risk DCIS dignosis.

	Non-RT					RT				
**Interval**	**O**	**E**	**RR**	**95% CI**	**P**	**O**	**E**	**RR**	**95% CI**	**P**
**0 - 1**	1	0.71	1.41	0.04-7.83	0.73	2	0.62	3.22	0.39-11.62	0.08
**1 - 5**	5	2.34	2.14	0.70-4.99	0.08	4	2.12	1.89	0.52-4.84	0.20
**5- 10**	2	1.75	1.14	0.14-4.12	0.85	4	1.64	2.44	0.66-6.24	0.07
**>10**	1	1.03	1.00	0.03-5.40	0.98	1	0.85	1.18	0.03-6.55	0.87

See below for legend.

Supplementary Table 17: Relative Risk-Time Course Specifics for Each Time Interval: NHL After Low/Intermediate-Risk DCIS dignosis.

	Non-RT					RT				
**Interval**	**O**	**E**	**RR**	**95% CI**	**P**	**O**	**E**	**RR**	**95% CI**	**P**
**0 - 1**	17	13.30	1.28	0.75-2.05	0.31	13	11.41	1.14	0.61-1.95	0.64
**1 - 5**	56	44.73	1.25	0.95-1.63	0.09	49	40.26	1.22	0.90-1.61	0.17
**5- 10**	31	34.47	0.90	0.61-1.28	0.55	42	32.60	1.29	0.93-1.74	0.10
**>10**	19	20.74	0.92	0.55-1.43	0.70	20	17.34	1.15	0.70-1.78	0.52

See below for legend.

Legend for Supplementary Tables 7-17: Abbreviations: O, observed SHM cases; E, expected SHM cases; RR, relative risk; CI, confidence interval.

Supplementary Table 18: Patient prognostic score[8]: risk stratification.

Distributions of prognostic scores based on risk factors			
**Score**	**Age (years)**	**Tumor Size (mm)**	**Grade**
**0**	61+	< 16	I
**1**	40-60	16-40	II
**2**	< 40	41+	III
**Total Points and risk group**			
**Low/intermediate risk**	0-2 scores		
**High risk**	3-6 scores		

Supplementary Table 19. Baseline Characteristics of Patients with Low/Intermediate-Risk DCIS by Radiotherapy.

Characteristic	Non-RT (n= 30,400)	RT (n= 28,465)	P
**Median age at DCIS diagnosis, (IQR), years**	64 (54, 73)	62 (54, 69)	**<.001[M]**
**Median year of DCIS diagnosis (IQR)**	2008 (2003, 2012)	2009 (2004, 2012)	**<.001[M]**
**Race**			
White	23,737 (78.1)	22,011 (77.3)	**<.001[χ]**
Black	2,963 (9.7)	3,044 (10.7)	
Others	3,700 (12.2)	3,410 (12.0)	
**Tumor size, mm**			
< 16	26,474 (87.1)	25,688 (90.2)	**<.001[χ]**
16-40	3,702 (12.2)	2,694 (9.5)	
41+	224 (0.7)	83 (0.3)	
**Grade**			
I	8,049 (26.5)	5,861 (20.6)	**<.001[χ]**
II	16,981 (55.9)	16,053 (56.4)	
III	5,370 (17.7)	6,551 (23.0)	
**ER**			
Negative	1,705 (5.6)	2,034 (7.1)	**<.001[χ]**
Positive	17,690 (58.2)	19,004 (66.8)	
Unknown	11,005 (36.2)	7,427 (26.1)	
**PR**			
Negative	3,011 (9.9)	3,610 (12.7)	**<.001[χ]**
Positive	14,966 (49.2)	16,100 (56.6)	
Unknown	12,423 (40.9)	8,755 (30.8)	
**Surgery**			
BCS	28,142 (98.9)	18,191 (59.8)	**<.001[χ]**
Mastectomy	207 (0.7)	10,715 (35.2)	
No/Unknown	116 (0.4)	1494 (4.9)	
**Median follow-up time of DCIS, years**	7.1 (3.7, 11.7)	7.2 (3.9, 11.4)	**.04[M]**
**Total person-years at risk**	244,600	227,091	

NOTE. Data presented as No. (%) unless otherwise stated where percentages were calculated within rows. P values were calculated using the χ^2 test (χ) and Mann-Whitney U tests (M).

Abbreviations: IQR, interquartile ratio; BCS, breast conserving surgery; RT, radiotherapy; DCIS, ductal carcinoma in situ; ER, estrogen receptor; PR, progesterone receptor status.

Supplementary Table 20. Univariable Competing Risk Regression Analysis of Risk of Developing Hematologic Malignancies in subjects with Low/Intermediate-Risk DCIS.

**Covariables**	**ALL**		**AML**		**CLL**		**CML**		**MM**		**HL**		**NHL**		**SHMs Combined**	
	**HR (95% CI)**	**P**	**HR (95% CI)**	**P**	**HR (95% CI)**	**P**	**HR (95% CI)**	**P**	**HR (95% CI)**	**P**	**HR (95% CI)**	**P**	**HR (95% CI)**	**P**	**HR (95% CI)**	**P**
**Age, per year**	**0.96 [0.93, 1.00]**	**0.07**	**1.05 [1.03, 1.08]**	**<.001**	**1.04 [1.02, 1.07]**	**<.001**	1.01 [0.98, 1.05]	0.40	**1.03[1.01, 1.04]**	**<.001**	0.98 [0.94, 1.03]	0.44	**1.03 [1.02, 1.04]**	**<.001**	**1.03 [1.03, 1.04]**	**<0.001**
**Year of diagnosis, per year**	0.93 [0.77, 1.13]	0.46	1.02 [0.95, 1.08]	0.64	0.98 [0.93, 1.02]	0.32	0.95 [0.87, 1.03]	0.20	0.99 [0.96, 1.03]	0.69	0.90 [0.84, 0.96]	.003	**0.96 [0.94, 0.99]**	**0.001**	**0.99 [0.98, 0.99]**	**<0.001**
**Race:** Black vs White	4.15 [0.75, 23.00]	0.10	1.39 [0.58, 3.31]	0.46	**0.30 [0.07, 1.22]**	**0.09**	0.52 [0.07, 3.90]	0.52	**2.92 [1.65, 5.17]**	**<.001**	/	/	0.77 [0.46, 1.28]	0.31	0.96 [0.79, 1.15]	0.64
**Race:** Others vs White	3.47 [0.63, 19.10]	0.15	0.39 [0.09, 1.62]	0.19	**0.25 [0.06, 1.01]**	**0.05**	0.44 [0.06, 3.28]	0.42	1.22 [0.57, 2.58]	0.61	/	/	0.91 [0.59, 1.40]	0.67	**0.74 [0.61, 0.92]**	**0.005**
**Tumor size**：16+ vs 1-16mm	1.26 [0.15, 10.03]	0.83	1.14 [0.45. 2.89]	0.79	0.97 [0.42, 2.26]	0.95	1.10 [0.25, 4.80]	0.90	0.69 [0.28, 1.72]	0.43	/	/	**3.14 [1.00, 5.21]**	**0.05**	0.98 [0.84, 1.15]	0.83
**Grade**：II vs I	0.43 [0.06, 3.03]	0.39	1.52 [0.66, 3.52]	0.32	1.04 [0.53, 2.04]	0.91	1.18 [0.37, 3.70]	0.78	0.69 [0.38, 1.25]	0.22	/	/	0.92 [0.65, 1.30]	0.63	0.92 [0.75, 1.14]	0.46
**Grade**：III vs I	2.31 [0.42, 12.60]	0.33	1.98 [0.78, 5.03]	0.15	**1.95 [0.95, 3.98]**	**0.07**	0.87 [0.20, 3.90]	0.86	1.49 [0.80, 2.75]	0.21	/	/	**1.72 [1.18, 2.48]**	**0.01**	1.06 [0.86, 1.30]	0.60
**ER**: positive vs negative	**0.18[0.03, 1.07]**	**0.06**	0.71 [0.25, 2.06]	0.53	0.50 [0.21, 1.23]	0.13	0.94 [0.12, 7.40]	0.95	1.27 [0.39, 4.13]	0.69	/	/	1.07 [0.57, 1.99]	0.84	0.84[0.68, 1.04]	0.11
**PR**: positive vs negative	0.36 [0.06, 2.16]	0.26	**0.45 [0.20, 1.02]**	**0.06**	**0.62 [0.30, 1.25]**	**0.06**	0.59 [0.11, 3.06]	0.53	0.72 [0.32, 1.60]	0.42	/	/	1.00 [0.64, 1.57]	0.37	**0.81 [0.67, 0.98]**	**0.03**
**Surgery:** mastectomy vs BCS	/	/	0.89 [0.40, 2.00]	0.78	1.17 [0.70, 5.19]	0.64	0.93 [0.27, 3.24]	0.91	1.05 [0.57, 1.92]	0.88	/	/	0.96 [0.67, 1.37]	0.82	**0.79 [0.68, 0.91]**	**0.01**
**Treatment**: RT vs Non-RT	*	/	0.91 [0.50, 1.66]	0.77	1.01 [0.61, 1.67]	0.97	1.38 [0.65, 3.50]	0.50	0.83 [0.52, 1.34]	0.44	1.84 [0.83, 7.78]	0.41	**1.16 [0.89, 1.52]**	**0.09**	**1.22 [1.09, 1.36]**	**<0.001**

NOTE. Shown are HRs and 95% CIs for developing a nonsynchronous (≥1 year after DCIS diagnosis) SHM in patients with DCIS, calculated using Fine-Gray competing risk regression analyses.

Bold type indicates the numbers that were significant in univariate analysis.

* There are 8 ALL cases after low/intermediate-risk DCIS in RT group, no one in Non-RT group. The regression failed to be converged.

Abbreviations: ALL, acute lymphocytic leukemia; AML, acute myeloid leukemia; CLL, chronic lymphocytic leukemia; CML, chronic myeloid leukemia; HL, Hodgkin lymphoma; HR, hazard ratio; MM, multiple myeloma; NHL, non-Hodgkin lymphoma; SHM, second hematologic malignancy; DCIS, ductal carcinoma in situ of the breast.

Supplementary Table 21. Multivariable Competing Risk Regression Analysis of Risk of Developing Hematologic Malignancies in subjects with Low/Intermediate-Risk DCIS.

Covariables	ALL		AML		CLL		CML		MM		HL		NHL		SHMs Combined	
	HR (95% CI)	P	HR (95% CI)	P	HR (95% CI)	P	HR (95% CI)	P	HR (95% CI)	P	HR (95% CI)	P	HR (95% CI)	P	HR (95% CI)	P
**Age, per year**	**0.96 [0.92, 0.99]**	**0.02**	**1.05 [1.02, 1.08]**	**<.0001**	**1.04 [1.02, 1.06]**	**<.001**	/	/	**1.03 [1.01, 1.04]**	**<.001**	/	/	**1.03 [1.02, 1.04]**	**<.001**	**1.03 [1.02, 1.04]**	**<.001**
**Year of diagnosis, per year**							/	/			/	/	**0.96 [0.93, 0.98]**	**<.001**	**0.96 [0.94, 0.98]**	**<.001**
**Race:** Black vs White					0.31 [0.08, 1.28]	0.11	/	/	**2.96 [1.68, 5.24]**	**<.001**	/	/			1.15 [0.84, 1.59]	0.38
**Race:** Others vs White					0.28 [0.07, 1.13]	0.08	/	/	1.31[0.62, 2.75]	0.48	/	/			0.86 [0.61, 1.20]	0.37
**Tumor size:** 16-40 vs 1-16mm							/	/			/	/	0.98 [0.63, 1.52]	0.90	0.99 [0.70, 1.40]	0.97
**Tumor size:** 41+ vs 1-16 mm							/	/			/	/	2.69 [0.85, 8.45]	0.09	1.94 [0.71, 5.34]	0.20
**Grade:** II vs I							/	/			/	/			0.99 [0.77, 1.28]	0.94
**Grade:** III vs I							/	/			/	/			**1.45 [1.08, 1.96]**	**0.02**
**ER**: positive vs negative	**0.14 [0.02, 0.81]**	**0.03**					/	/			/	/			1.57 [0.99, 2.50]	0.06
**PR**: positive vs negative			0.53 [0.24, 1.18]	0.12	0.58 [0.27, 1.24]	0.16	/	/			/	/			**0.68 [0.47, 0.98]**	**0.04**
**Surgery:** mastectomy vs BCS							/	/			/	/			1.11 [0.82, 1.50]	0.49
**Treatment**: RT vs Non-RT							/	/			/	/	**1.32 [1.00, 1.73]**	**0.048**	1.24 [0.98, 1.55]	0.07

NOTE. Shown are HRs and 95% CIs for developing a nonsynchronous (⩾1 year after DCIS diagnosis) SHM in patients with DCIS, calculated using Fine-Gray competing risk regression analyses. Covariables that were significant in univariable analyses ($P < 0.1$) were included in the multivariable analysis, which was subjected to the backward selection procedure to generate the final model.

The large sample size of the SHMs combined analysis allowed for inclusion of all covariables in the multivariable model, which was also subjected to a backward selection procedure. Univariable regression analyses are shown in the Data Supplement. Bold type indicates the numbers that remained significant in multivariate analysis.

Abbreviations: ALL, acute lymphocytic leukemia; AML, acute myeloid leukemia; CLL, chronic lymphocytic leukemia; CML, chronic myeloid leukemia; HL, Hodgkin lymphoma; HR, hazard ratio; MM, multiple myeloma; NHL, non-Hodgkin lymphoma; SHM, second hematologic malignancy; DCIS, ductal carcinoma in situ of the breast.

Supplementary Table 22: Characteristics of DCIS Cases and Controls.

Parameter		DCIS cases that later developed ALL				DCIS cases that later developed CML				DCIS cases that later developed NHL			
		Non-RT group		RT group		Non-RT group		RT group		Non-RT group		RT group	
		Cases (n =7)	Controls (n = 35)	Cases (n = 19)	Controls (n =95)	Cases (n = 25)	Controls (n = 125)	Cases (n = 26)	Controls (n = 130)	Cases (n = 419)	Controls (n = 2,095)	Cases (n = 298)	Controls (n = 1,490)
Age (in years, median [IQR])		61(49-65)	60(48-66)	61(50-64)	59(46-62)	66(46-74)	62(52-72)	58(51-62)	57(51-67)	66(55-74)	65(54-75)	64(57-71)	64(55-72)
Median year of DCIS diagnosis (IQR)		1994 (1992-2002)	1996 (1989-2008)	2000 (1997-2007)	2003 (1998-2008)	2004 (1995-2008)	2002 (1995-2010)	2004 (2002-2008)	2004 (2000-2009-)	2001 (1995-2006)	2001 (1995-2006)	2003 (2000-2007)	2005 (2000-2009)
Race	White	7(100.0)	33 (94.3)	13 (68.4)	62 (65.3)	22 (88.0)	115 (92.0)	23 (88.5)	122 (93.8)	355 (84.7)	1772 (84.6)	251 (84.2)	1253 (84.1)
	Black	0 (0.0)	0 (0.0)	3 (15.8)	16 (16.8)	2 (8.0)	8 (6.4)	2 (7.7)	3 (2.3)	27 (6.4)	128 (6.1)	25 (8.4)	123 (8.3)
	Other	0 (0.0)	2 (5.7)	3 (15.8)	17 (17.9)	1 (4.0)	2 (1.6)	1 (3.8)	5 (3.8)	37 (8.8)	195 (9.3)	22 (7.4)	114 (7.7)
Tumor size	<2 cm	1 (14.3)	9 (25.7)	12 (63.2)	57 (60.0)	10 (40.0)	46 (36.8)	15 (57.7)	76 (58.5)	208 (49.6)	1063 (50.7)	187 (62.8)	947 (63.6)
	2-5 cm	0 (0.0)	3 (8.6)	3 (15.8)	16 (16.8)	5 (20.0)	33 (26.4)	6 (23.1)	27 (20.8)	45 (10.7)	219 (10.5)	36 (12.1)	159 (10.7)
	≥5 cm	1 (14.3)	6 (17.1)	0 (0.0)	0 (0.0)	3 (12.0)	11 (8.8)	1 (3.8)	7 (5.4)	16 (3.8)	76 (3.6)	7 (2.3)	36 (2.4)
	Unknown	5 (71.4)	17 (48.6)	4 (21.1)	22 (23.2)	7 (28.0)	35 (28.0)	4 (15.4)	20 (15.4)	150 (35.8)	737 (35.2)	68 (22.8)	348 (23.4)
Grade	I	0 (0.0)	0 (0.0)	2 (10.5)	12 (12.6)	2 (8.0)	9 (7.2)	3 (11.5)	9 (6.9)	45 (10.7)	226 (10.8)	24 (8.1)	132 (8.9)
	II	1 (14.3)	4 (11.4)	4 (21.1)	26 (27.4)	8 (32.0)	38 (30.4)	10 (38.5)	55 (42.3)	94 (22.4)	437 (20.9)	86 (28.9)	435 (29.2)
	III	1 (14.3)	9 (25.7)	9 (47.4)	38 (40.0)	6 (24.0)	33 (26.4)	8 (30.8)	38 (29.2)	118 (28.2)	679 (32.4)	101 (33.9)	529 (35.5)
	Unknown	5 (71.4)	22 (62.9)	4 (21.1)	19 (20.0)	9 (36.0)	45 (36.0)	5 (19.2)	28 (21.5)	162 (38.7)	753 (35.9)	87 (29.2)	394 (26.4)
ER status	Negative	1 (14.3)	4 (11.4)	3 (15.8)	12 (12.6)	2 (8.0)	11 (8.8)	5 (19.2)	17 (13.1)	27 (6.4)	139 (6.6)	28 (9.4)	169 (11.3)
	Positive	1 (14.3)	12 (34.3)	6 (31.6)	37 (38.9)	9 (36.0)	48 (38.4)	9 (34.6)	50 (38.5)	117 (27.9)	515 (24.6)	132 (44.3)	689 (46.2)

	Unknown	5 (71.4)	19 (54.3)	10 (52.6)	46 (48.4)	14 (56.0)	66 (52.8)	12 (46.2)	63 (48.5)	275 (65.6)	1441 (68.8)	138 (46.3)	632 (42.4)
PR status	Negative	1 (14.3)	7 (20.0)	3 (15.8)	11 (11.6)	2 (8.0)	14 (11.2)	6 (23.1)	25 (19.2)	46 (11.0)	237 (11.3)	43 (14.4)	258 (17.3)
	Positive	1 (14.3)	8 (22.9)	6 (31.6)	35 (36.8)	5 (20.0)	24 (19.2)	8 (30.8)	42 (32.3)	85 (20.3)	356 (17.0)	108 (36.2)	560 (37.6)
	Unknown	5 (71.4)	20 (57.1)	10 (52.6)	49 (51.6)	18 (72.0)	87 (69.6)	12 (46.2)	63 (48.5)	288 (68.7)	1502 (71.7)	147 (49.3)	672 (45.1)
Surgery	Non-surgery	0 (0.0)	0 (0.0)	0 (0.0)	0 (0.0)	9 (36.0)	36 (28.8)	26 (100.0)	130 (100.0)	15 (3.6)	81 (3.9)	2 (0.7)	14 (0.9)
	Breast conserving surgery	4 (57.1)	22 (62.9)	19 (100.0)	95 (100.0)	9 (36.0)	53 (42.4)	0 (0.0)	0 (0.0)	189 (45.1)	928 (44.3)	290 (97.3)	1440 (96.6)
	Mastectomy	1 (14.3)	8 (22.9)	0 (0.0)	0 (0.0)	7 (28.0)	36 (28.8)	0 (0.0)	0 (0.0)	133 (31.7)	691 (33.0)	4 (1.3)	28 (1.9)
	Unknown	2 (28.6)	5 (14.3)	0 (0.0)	0 (0.0)	0 (0.0)	0 (0.0)	0 (0.0)	0 (0.0)	82 (19.6)	395 (18.9)	2 (0.7)	8 (0.5)
Median PYs of follow-up (IQR)		10.9	11.1	8.2	11.6	9.2 (6.1-14.2)	11.1(5.3-16.0)	11.1(6.5-14.1)	11.7(6.7-15.1)	11.3(6.1-16.5)	12.0(6.8-16.9)	10.7(7.0-14.4)	10.5(6.3-15.1)
Total PYs of follow-up		110	513	153	1187	268	1413	275	1467	5059	26463	3230	16547

Entries were used for the case-control survival analyses shown in Figure 3 of the main article. Abbreviations: PY, person-year; RT, radiotherapy; ALL, acute lymphocytic leukemia; CML, chronic myeloid leukemia; NHL, non-Hodgkin lymphoma; DCIS, ductal carcinoma in situ of the breast.

Supplementary Table 23: Characteristics of ALL, CML and NHL Cases and Controls.

Parameter		ALL cases after treatment for DCIS				CML cases after treatment for DCIS				NHL cases after treatment for DCIS			
		Non-RT group		RT group		Non-RT group		RT group		Non-RT group		RT group	
		Cases (n =7)	Controls (n = 35)	Cases (n = 19)	Controls (n =95)	Cases (n = 25)	Controls (n = 125)	Cases (n = 26)	Controls (n = 130)	Cases (n = 419)	Controls (n = 2,095)	Cases (n = 298)	Controls (n = 1,490)
Age (in years, median [IQR])		67(54-83)	73(15-83)	63(56-69)	65(52-77)	69(55-80)	70(56-82)	64(58-69)	64(53-76)	73(63-81)	73(62-81)	71(64-78)	70(59-79)
Median year of DCIS diagnosis (IQR)		2007 (2004-2011)	2005 (1999-2011)	2009 (2003-2012)	2008 (2002-2013)	2008 (2005-2013)	2008 (2003-2013)	2012 (2008-2013)	2012 (2009-2012)	2008 (2002-2012)	2008 (2003-2012)	2010 (2006-2014)	2009 (2004-2013)
Race	White	7 (100.0)	33 (94.3)	13 (68.4)	66 (69.5)	22 (88.0)	114 (91.2)	23 (88.5)	111 (85.4)	355 (84.7)	1779 (84.9)	251 (84.2)	1251 (84.0)
	Black	0 (0.0)	1 (2.9)	3 (15.8)	12 (12.6)	2 (8.0)	10 (8.0)	2 (7.7)	15 (11.5)	27 (6.4)	132 (6.3)	25 (8.4)	137 (9.2)
	Other	0 (0.0)	1 (2.9)	3 (15.8)	17 (17.9)	1 (4.0)	1 (0.8)	1 (3.8)	4 (3.1)	37 (8.8)	184 (8.8)	22 (7.4)	102 (6.8)
Treated with chemotherapy		3 (42.9)	15 (42.9)	15 (78.9)	78 (82.1)	15 (60.0)	74 (59.2)	19 (73.1)	97 (74.6)	218 (52.0)	1111 (53.0)	160 (53.7)	836 (56.1)
Median PYs of follow-up (IQR)		2.7(1.7-4.1)	0.7(0.2-5.2)	1.4(0.6-3.9)	0.8(0.2-2.0)	3.6(2.0-7.5)	2.9(0.8-6.5)	3.8(2.0-5.5)	2.5(1.0-4.9)	3.9(1.0-8.0)	3.0(0.6-7.0)	2.9(1.0-6.7)	3.0(0.7-7.0)
Total PYs of follow-up		49.5	168.2	53.5	190.8	122.0	553.5	117.1	416.0	2276.7	9720.9	1284.7	6882.0

Entries were used for the case-control survival analyses shown in Figure 3 of the main article. Abbreviations: PY, person-year; RT, radiotherapy; ALL, acute lymphocytic leukemia; CML, chronic myeloid leukemia; NHL, non-Hodgkin lymphoma; DCIS, ductal carcinoma in situ of the breast.

Supplementary Table 24: Histologic Characteristics of ALL Cases and Controls.

ICD-O-3 code	Histologic subtype	Non-RT group		RT group	
		Cases (n =7)	Controls (n = 35)	Cases (n = 19)	Controls (n =95)
9811	B lymphoblastic leukemia/lymphoma, NOS	0 (0.0)	0 (0.0)	4 (21.1)	22 (23.2)
9820	Lymphoid leukemia, NOS	1 (14.3)	4 (11.4)	1 (5.3)	4 (4.2)
9827	Adult T-cell leukemia/lymphoma (HTLV-1 pos.)	0 (0.0)	0 (0.0)	1 (5.3)	7 (7.4)
9834	Prolymphocytic leukemia, T-cell type	2 (28.6)	11 (31.4)	3 (15.8)	8 (8.4)
9835	Precursor cell lymphoblastic leukemia, NOS	4 (57.1)	20 (57.1)	5 (26.3)	30 (31.6)
9836	Precursor B-cell lymphoblastic leukemia	0 (0.0)	0 (0.0)	3 (15.8)	13 (13.7)
9837	T lymphoblastic leukemia/lymphoma	0 (0.0)	0 (0.0)	2 (10.5)	11 (11.6)

Entries were used for the case-control survival analyses shown in Figure 3 of the main article. Percentages shown are calculated within rows. Abbreviations: ALL, acute lymphocytic leukemia; NOS, not otherwise specified; RT, radiotherapy.

Supplementary Table 25: Histologic Characteristics of CML Cases and Controls.

ICD-O-3 code	Histologic subtype	Non-RT group		RT group	
		Cases (n = 25)	Controls (n = 125)	Cases (n = 26)	Controls (n = 130)
9875	Chronic myelogenous leukemia, BCR/ABL positive	8 (32.0)	37 (29.6)	12 (46.2)	57 (43.8)
9863	Chronic myeloid leukemia, NOS	17 (68.0)	88 (70.4)	14 (53.8)	73 (56.2)

Entries were used for the case-control survival analyses shown in Figure 3 of the main article. Percentages shown are calculated within rows. Abbreviations: CML, chronic myeloid leukemia; NOS, not otherwise specified; RT, radiotherapy.

Supplementary Table 26: Histologic Characteristics of NHL Cases and Controls.

ICD-O-3 code	Histologic subtype	Non-RT group		RT group	
		Cases (n = 419)	Controls (n = 2,095)	Cases (n = 298)	Controls (n = 1,490)
9590	Malignant lymphoma, NOS	13 (3.1)	63 (3.0)	8 (2.7)	45 (3.0)
9591	Malignant lymphoma, non-Hodgkin	36 (8.6)	185 (8.8)	30 (10.1)	149 (10.0)
9596	Composite Hodgkin and non-Hodgkin lymphoma	0 (0.0)	0 (0.0)	1 (0.3)	2 (0.1)
9597	Primary Cutaneous follicle centre lymphoma	0 (0.0)	0 (0.0)	2 (0.7)	7 (0.5)
9671	ML, lymphoplasmacytic	7 (1.7)	48 (2.3)	7 (2.3)	34 (2.3)
9673	Mantle cell lymphoma	16 (3.8)	73 (3.5)	13 (4.4)	59 (4.0)
9675	ML, mixed sm. and lg. cell, diffuse	2 (0.5)	6 (0.3)	1 (0.3)	8 (0.5)
9679	Mediastinal large B-cell lymphoma	0 (0.0)	0 (0.0)	0 (0.0)	1 (0.1)
9680	ML, large B-cell, diffuse	141 (33.7)	735 (35.1)	105 (35.2)	538 (36.1)
9684	ML, large B-cell, diffuse, immunoblastic, NOS	1 (0.2)	8 (0.4)	2 (0.7)	10 (0.7)
9687	Burkitt lymphoma, NOS	0 (0.0)	0 (0.0)	0 (0.0)	2 (0.1)
9689	Splenic marginal zone B-cell lymphoma	3 (0.7)	15 (0.7)	10 (3.4)	26 (1.7)
9690	Follicular lymphoma, NOS	29 (6.9)	158 (7.5)	17 (5.7)	71 (4.8)
9691	Follicular lymphoma, grade 2	30 (7.2)	134 (6.4)	16 (5.4)	80 (5.4)
9695	Follicular lymphoma, grade 1	36 (8.6)	172 (8.2)	16 (5.4)	80 (5.4)

9698	Follicular lymphoma, grade 3	25 (6.0)	115 (5.5)	8 (2.7)	43 (2.9)
9699	Marginal zone B-cell lymphoma, NOS	50 (11.9)	237 (11.3)	31 (10.4)	155 (10.4)
9700	T-CELL LYMPHOMAS, mycosis fungoides	8 (1.9)	31 (1.5)	10 (3.4)	65 (4.4)
9702	Mature T-cell lymphoma, NOS	9 (2.1)	42 (2.0)	5 (1.7)	31 (2.1)
9705	Angioimmunoblastic T-cell lymphoma	1 (0.2)	5 (0.2)	5 (1.7)	17 (1.1)
9709	Cutaneous T-cell lymphoma, NOS	5 (1.2)	36 (1.7)	4 (1.3)	37 (2.5)
9714	Anaplastic large cell lymphoma, T-cell and Null cell type	6 (1.4)	25 (1.2)	3 (1.0)	14 (0.9)
9718	Primary cutan. CD30+ T-cell lymphoprolif. Disorder	1 (0.2)	7 (0.3)	2 (0.7)	8 (0.5)
9719	NK/T-cell lymphoma, nasal and nasal-type	0 (0.0)	0 (0.0)	2 (0.7)	4 (0.3)
9727	Precursor cell lymphoblastic lymphoma, NOS	0 (0.0)	0 (0.0)	0 (0.0)	3 (0.2)
9728	Precursor B-cell lymphoblastic lymphoma	0 (0.0)	0 (0.0)	0 (0.0)	1 (0.1)

Entries were used for the case-control survival analyses shown in Figure 3 of the main article. Percentages shown are calculated within rows. Abbreviations: NHL, non-Hodgkin lymphoma; NOS, not otherwise specified; RT, radiotherapy.

Supplementary Table 27. SIRs of Second Hematologic Malignancies in Patients With DCIS diagnosed from 2001 to 2016.

SHMs	Non-RT				RT				Additional Risk From RT	
	Observed	Expected	SIR (95% CI)	P	Observed	Expected	SIR (95% CI)	P	SIR (95% CI)	P
**SHMs combined**	369	370	100 (90-110)	0.96	367	**316**	**116(105-128)**	**0.004**	**116(105, 129)**	**0.004**
**ALL**	3	6	47(1-139)	0.22	8	6	145(63-286)	0.41	309 (57, 560)	0.14
**AML**	40	38	105(75-143)	0.75	34	32	106(73-148)	0.72	100 (75, 143)	0.63
**CLL**	40	55	72(52-99)	0.04	50	46	108(80-143)	0.56	150 (81, 215)	0.07
**CML**	15	10	153(86-252)	0.11	21	8	**250(155-382)**	**<0.001**	**163 (102, 247)**	**<0.001**
**MM**	75	71	106(83-133)	0.63	64	61	104(80-133)	0.70	98 (83, 132)	0.54
**HL**	4	10	40(11-102)	0.06	8	9	91(39-179)	0.74	228 (53, 403)	0.06
**NHL**	192	180	107(92-123)	0.37	182	154	**119(102-137)**	**0.02**	**110 (1.01, 1.23)**	**0.02**

NOTE. Not including second malignant neoplasms that occurred in the first year after DCIS diagnosis. An SIR of 100 indicates a similar ratio as the background population.

Abbreviations: DCIS, ductal carcinoma in situ; ALL, acute lymphocytic leukemia; AML, acute myeloid leukemia; CLL, chronic lymphocytic leukemia; CML, chronic myeloid leukemia; MM, multiple myeloma; HL, Hodgkin lymphoma; NHL, non-Hodgkin lymphoma; HR, hazard ratio; RT, radiotherapy; SHM, second hematologic malignancy; SIR, standardized incidence ratio.

Supplementary Table 28. SIRs of Second Hematologic Malignancies in Patients With DCIS diagnosed from 1975 to 2000.

SHMs	Non-RT				RT				Additional Risk From RT	
	Observed	Expected	SIR (95% CI)	P	Observed	Expected	SIR (95% CI)	P	SIR (95% CI)	P
**SHMs combined**	358	392	91 (82, 101)	0.09	195	168	**116 (100-134)**	**0.04**	**127 (1.01-1.33)**	**0.007**
**ALL**	4	6	66 (18-169)	0.41	9	3	**341 (156-646)**	**<0.001**	**517 (346-688)**	**<0.001**
**AML**	32	39	81 (55-114)	0.26	24	17	143 (92-213)	0.09	176 (98-220)	0.06
**CLL**	76	63	121 (96-152)	0.10	29	26	111 (74-160)	0.56	92 (75-150)	0.08
**CML**	7	11	66 (27-136)	0.23	3	4	69 (14-201)	0.62	105 (80-131)	0.19
**MM**	62	74	84 (65-108)	0.16	35	32	110 (77-154)	0.60	131 (76-152)	0.14
**HL**	13	10	127 (68-217)	0.34	5	5	110 (36-257)	0.99	87 (32, 222)	0.34
**NHL**	164	189	87 (74-109)	0.07	90	82	110 (88-135)	0.38	**126 (101, 135)**	**0.04**

NOTE. Not including second malignant neoplasms that occurred in the first year after DCIS diagnosis. An SIR of 100 indicates a similar ratio as the background population.

Abbreviations: DCIS, ductal carcinoma in situ; ALL, acute lymphocytic leukemia; AML, acute myeloid leukemia; CLL, chronic lymphocytic leukemia; CML, chronic myeloid leukemia; MM, multiple myeloma; HL, Hodgkin lymphoma; NHL, non-Hodgkin lymphoma; HR, hazard ratio; RT, radiotherapy; SHM, second hematologic malignancy; SIR, standardized incidence ratio.

Supplemental References

1. National Cancer Institute: Surveillance, Epidemiology, and End Results Program. (http://seer.cancer.gov/).
2. Radivoyevitch T, Sachs RK, Gale RP, et al. Defining AML and MDS second cancer risk dynamics after diagnoses of first cancers treated or not with radiation. *Leukemia.* 2016;30(2):285-294.
3. Computing RFfS. R Core Team: R: A language and environment for statistical computing. 2017.
4. Hastie T, Tibshirani R. Generalized additive models for medical research. *R Foundation for Statistical Computing.* 1995;4(3):187-196.
5. Scrucca L, Santucci A, Aversa F. Regression modeling of competing risk using R: an in depth guide for clinicians. *Bone marrow transplantation.* 2010;45(9):1388-1395.
6. Li H, Han D, Hou Y, Chen H, Chen Z. Statistical inference methods for two crossing survival curves: a comparison of methods. *PloS one.* 2015;10(1):e0116774.
7. Qiu P, Sheng J. A Two-Stage Procedure for Comparing Hazard Rate Functions. *Journal of the Royal Statistical Society Series B (Statistical Methodology).* 2008;70(1):191-208.
8. Smith GL, Smith BD, Haffty BG. Rationalization and regionalization of treatment for ductal carcinoma in situ of the breast. *International journal of radiation oncology, biology, physics.* 2006;65(5):1397-1403.
